# Comorbidity of asthma and attention deficit hyperactivity disorder in childhood: causal, shared early-life risk factors, or shared genetic liability?

**DOI:** 10.1093/ije/dyag074

**Published:** 2026-05-23

**Authors:** Mohammad Talaei, Panagiota Pagoni, Evie Stergiakouli, Seif O Shaheen

**Affiliations:** Centre for Preventive Neurology, Wolfson Institute of Population Health, Queen Mary University of London, London, EC1M 6BQ, United Kingdom; MRC Integrative Epidemiology Unit, University of Bristol, Bristol, BS1 5DS, United Kingdom; School of Psychology, University of Exeter, Exeter, EX4 4QG, United Kingdom; MRC Integrative Epidemiology Unit, University of Bristol, Bristol, BS1 5DS, United Kingdom; Population Health Sciences, Bristol Medical School, University of Bristol, Bristol, BS8 2PS, United Kingdom; Centre for Preventive Neurology, Wolfson Institute of Population Health, Queen Mary University of London, London, EC1M 6BQ, United Kingdom; Allergy and Lung Health Unit, Melbourne School of Population and Global Health, The University of Melbourne, Melbourne, VIC 3052, Australia

**Keywords:** ADHD, asthma, genetic risk, early life, risk factor, causal inference, childhood, ALSPAC

## Abstract

**Background:**

The positive association between childhood asthma and attention deficit hyperactivity disorder (ADHD) has not been adequately explained. We aimed to investigate whether it is causal or whether shared genetics and/or shared early-life risk factors explain the link.

**Methods:**

We used a two-sample Mendelian randomization (MR) to test whether there is a causal relationship between asthma and ADHD. In the Avon Longitudinal Study of Parents and Children (ALSPAC), we defined doctor-diagnosed asthma, its endotypes according to atopy determined by skin-prick tests, and the risk of ADHD by using the Strengths and Difficulties Questionnaire, all at 7 years of age. We explored whether the asthma–ADHD association was attenuated when controlling for an extensive number of early-life risk factors (*N* = 7165). Child polygenic risk scores (PRSs) for asthma and ADHD were calculated by using published genome-wide association studies across seven *P*-value thresholds (<5 × 10^−8^ to <.5) (*N* = 5425–5503).

**Results:**

We found little evidence of a causal effect between asthma and ADHD in both directions. In ALSPAC, asthma [odds ratio (OR) 1.35, 95% confidence interval (CI) 1.10 − 1.65], particularly non-atopic asthma (OR 1.51, 95% CI 1.09 − 2.08), was associated with ADHD in crude models, while atopic asthma was not (OR 1.00, 95% CI 0.69 − 1.45). These associations were largely attenuated after adjusting for 15 shared early-life risk factors (OR 1.14, 95% CI 0.92 − 1.41 for asthma and OR 1.24, 95% CI 0.88 − 1.74 for non-atopic asthma). There was little evidence of association between asthma PRSs and ADHD at any *P*-value threshold. We found evidence of an association between ADHD PRSs and asthma and non-atopic asthma at most thresholds; the strongest association was at *P *< 5 × 10^−3^ (OR 1.12, 95% CI 1.04 − 1.22, *P *= .004) for asthma and at *P *< 5 × 10^−2^ (OR 1.18, 95% CI 1.04 − 1.34, *P *= .01) for non-atopic asthma.

**Conclusion:**

The association between asthma and ADHD in childhood is unlikely to be causal and is largely explained by shared early-life risk factors, with some evidence for a shared genetic background.

Key Messages

- We evaluated evidence for three potential explanations for the association between asthma and attention deficit hyperactivity disorder (ADHD) in children: (i) causal, (ii) explained by shared early-life risk factors, and/or (iii) explained by shared genetics.
- The asthma–ADHD association is unlikely to be causal, but shared genetic liability to ADHD and shared early-life risk factors, particularly socioeconomic status and maternal anxiety during pregnancy, may partly explain the association in mid-childhood.
- This study has enhanced our understanding of the etiological factors underlying asthma–ADHD comorbidity. Future studies should investigate the role of maternal anxiety in pregnancy in the development of both conditions in offspring.

## Introduction

Children with asthma are more likely to suffer from attention deficit hyperactivity disorder (ADHD) [1–3], but the reason for the frequent coexistence of these common chronic disorders is unknown. The asthma–ADHD association could partly reflect a causal effect of one condition on the other. This was previously explored by using a Mendelian randomization (MR) approach [4]. The evidence to date, however, is conflicting; one study suggested a small causal effect of ADHD on asthma (not vice versa) [5], but this was not confirmed by others [6, 7]. Another possible explanation is that both conditions share genetic causes, but this has been investigated in only a few studies, with conflicting results [5, 8].

An alternative explanation is that the asthma–ADHD association is explained by shared early-life risk factors. Asthma and ADHD have many prenatal and perinatal risk factors in common, but their association persisted when previous studies controlled for some of these [1, 2, 9, 10]. However, other potential shared maternal risk factors that might explain this comorbidity have not been controlled for previously. These include paracetamol use [11, 12], antibiotic use [13, 14], stress [15, 16], sugar intake [17, 18], pre-eclampsia [19, 20], and obesity [21, 22]. There is little evidence that maternal smoking in pregnancy causes ADHD [23]. However, maternal genetic liability to ADHD was associated with some risk factors for child ADHD, raising the potential for genetic confounding [24]. There is evidence that ADHD is also positively associated with atopic phenotypes and biomarkers [3, 25–28], suggesting possible shared immunological mechanisms.

While these potential explanations for the asthma–ADHD association are not mutually exclusive, identifying shared genetic or environmental risk factors could advance our understanding of underlying mechanisms, with implications for primary prevention. In this study, we investigated whether (i) shared early-life risk factors or (ii) shared genetics could explain the associations between asthma and other atopic phenotypes and ADHD and (iii) explored the evidence for one condition causing the other.

## Methods

### Study population

The Avon Longitudinal Study of Parents and Children (ALSPAC) is a prospective birth cohort study based in Bristol, UK and surrounding areas. Pregnant women with expected delivery dates of between 1 April 1991 and 31 December 1992 were recruited; 14 541 women were initially enrolled, with 14 062 children born and 13 988 children alive at 1 year of age. Participants have been followed up since birth with questionnaires and objective measures in research clinics. Detailed information about the cohort can be found elsewhere [29, 30] in addition to a fully searchable data dictionary and variable search tool (http://www.bristol.ac.uk/alspac/researchers/our-data/).

### Outcome assessments

We defined current doctor-diagnosed asthma at ∼7 years (91 months) of age, which was in good agreement with GP-recorded diagnoses (sensitivity 88.5%, specificity 95.7%) [31]. A skin-prick allergy test was performed at age 7 years to define atopy [32]. We categorized participants into four mutually exclusive endotypes: none, atopy-only, atopic asthma, and non-atopic asthma. Current eczema and hay fever in children at age 7 years were similarly defined by parental reports.

The Strengths and Difficulties Questionnaire (SDQ; http://www.sdqinfo.org) [33] is a behavioral screening tool known for its high reliability and validity in detecting psychiatric diagnoses [34]. One subscale of the SDQ evaluates symptoms of combined hyperactive–impulsive (three items) and inattentive (two items) ADHD. This subscale shows minimal overlap with other SDQ factors and excellent discrimination for parent reports in the general population [35]. We used mother reports obtained at ∼7 years (81 months) of child age (and 9 years for sensitivity analysis) and defined ADHD as a score of ≥7 [33].

### Early-life risk factors

**Figure 1:**
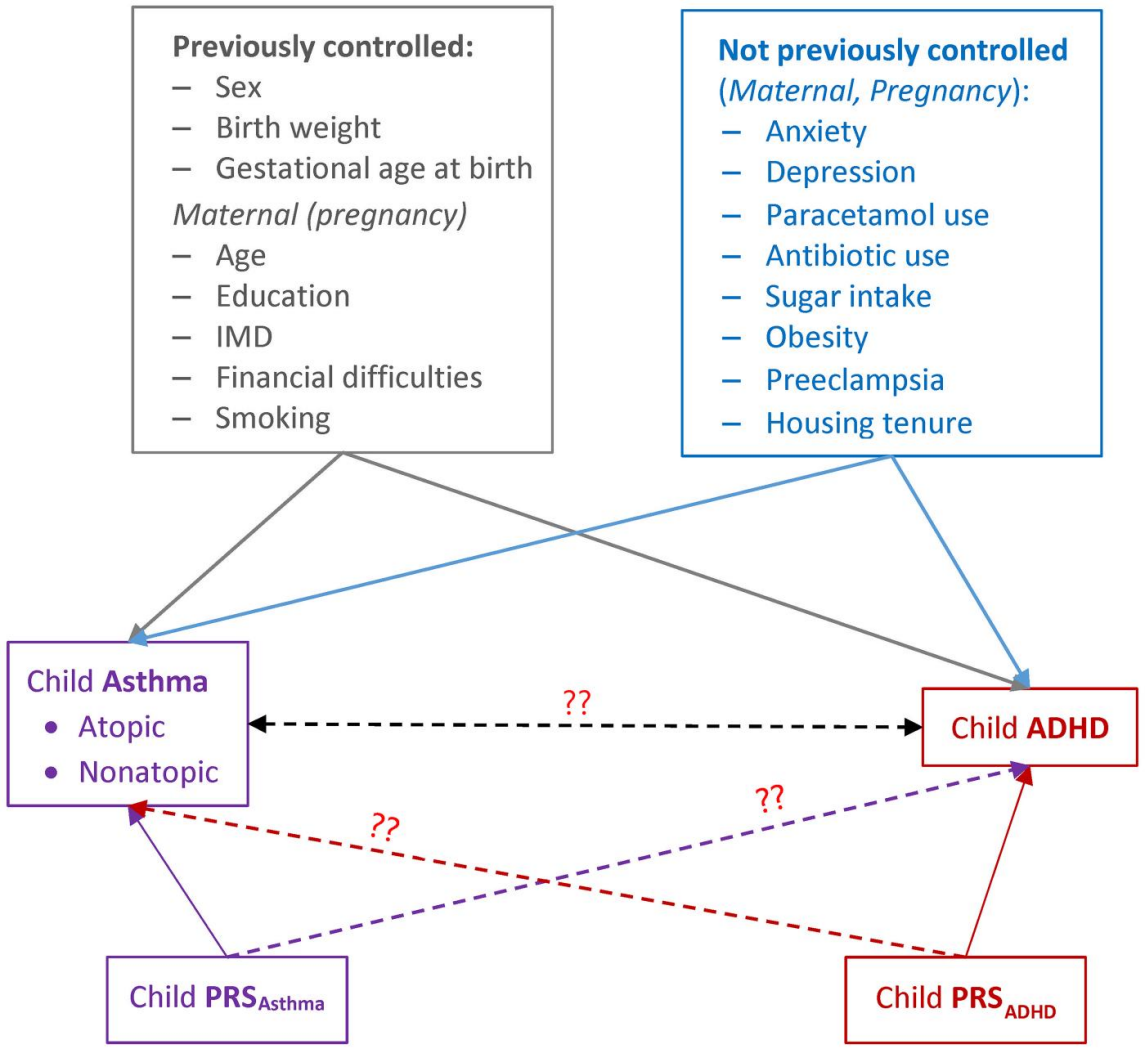
Conceptual model of shared genetic and early-life risk factors underlying the association between asthma and ADHD. PRS, polygenic risk score; IMD, index of multiple deprivation.

We reviewed the existing literature to identify early-life risk factors (maternal/prenatal/perinatal) that previous studies of the asthma–ADHD association had controlled for and those that have been reported to be associated separately with asthma and ADHD but were not controlled for in previous studies. Additional socioeconomic factors, such as housing tenure and financial difficulties at birth, were considered (Fig. 1). We used the directed acyclic graph approach [36] to explore these factors further (Supplementary Figure 1). Rather than just eliminating the confounding effect, we were more interested in identifying confounding due to shared risk factors, to better understand the mechanisms underlying asthma–ADHD comorbidity.

### Polygenic risk scores

We used the most recently published genome-wide association study (GWAS) summary data for asthma (*N*_cases_/*N*_controls_ = 23 948/118 538) [37] and ADHD (*N*_cases_/*N*_controls_ = 38 691/186 843) [38], which included samples from children and adults (the discovery data; Supplementary Table 1). We prioritized GWASs that included childhood samples to ensure relevance for our study of childhood-onset traits, even though this results in some overlap with the ALSPAC cohort (the target data). Larger GWASs exist in some cases, but these are based on adult populations and may not adequately capture genetic influences in children.

Before calculating the polygenic risk scores (PRSs), we excluded ambiguous single-nucleotide polymorphisms (SNPs) and SNPs located in the Major Histocompatibility Complex region (chr: 6; 29–34 Mb) due to its complex linkage disequilibrium structure. This region was excluded following the standard practice in PRS analyses, as its inclusion can lead to unstable effect estimates and inflated scores. Also, its exclusion has a negligible impact on asthma PRS performance [37]. Additionally, SNPs with mismatching alleles between discovery and target datasets were excluded. Using ALSPAC as the reference panel, SNPs were clumped by using an *r*^2^ of 0.25 and a physical distance threshold of 500 kb. We calculated PRSs and standardized them across seven *P*-value thresholds (*P* < 5 × 10^−8^ to *P* <.5) (Supplementary Tables 2 and 3, and Supplementary Figure 2), as their power depends on the discovery and target sample sizes and SNP inclusion parameters [38, 39].

### Two-sample MR

MR is an observational method that relies on an instrumental variable (IV) approach, using genetic variants as proxies for exposures to estimate causal effects under certain assumptions. We applied two-sample MR, in which the instrument–exposure and instrument–outcome effect sizes and standard errors are extracted from independent GWASs of the same underlying population [40]. The GWASs identified as discovery for the PRS calculations were used for the extraction of genetic instruments (Supplementary Table 1). Approximately independent genetic variants were identified (*r*^2^ < 0.01 within a 10 000-kb window, *P* <5 × 10^−8^) and corresponding log odds ratios (ORs) and standard errors were extracted from summary-level datasets. Genetic variants used as proxies for exposure were then extracted from the outcome GWAS (or proxy variants if not present) and the data were harmonized. As in any MR analysis, we made the three core IV assumptions (relevance, independence, and exclusion restriction).

### Statistical analysis

**Figure 2:**
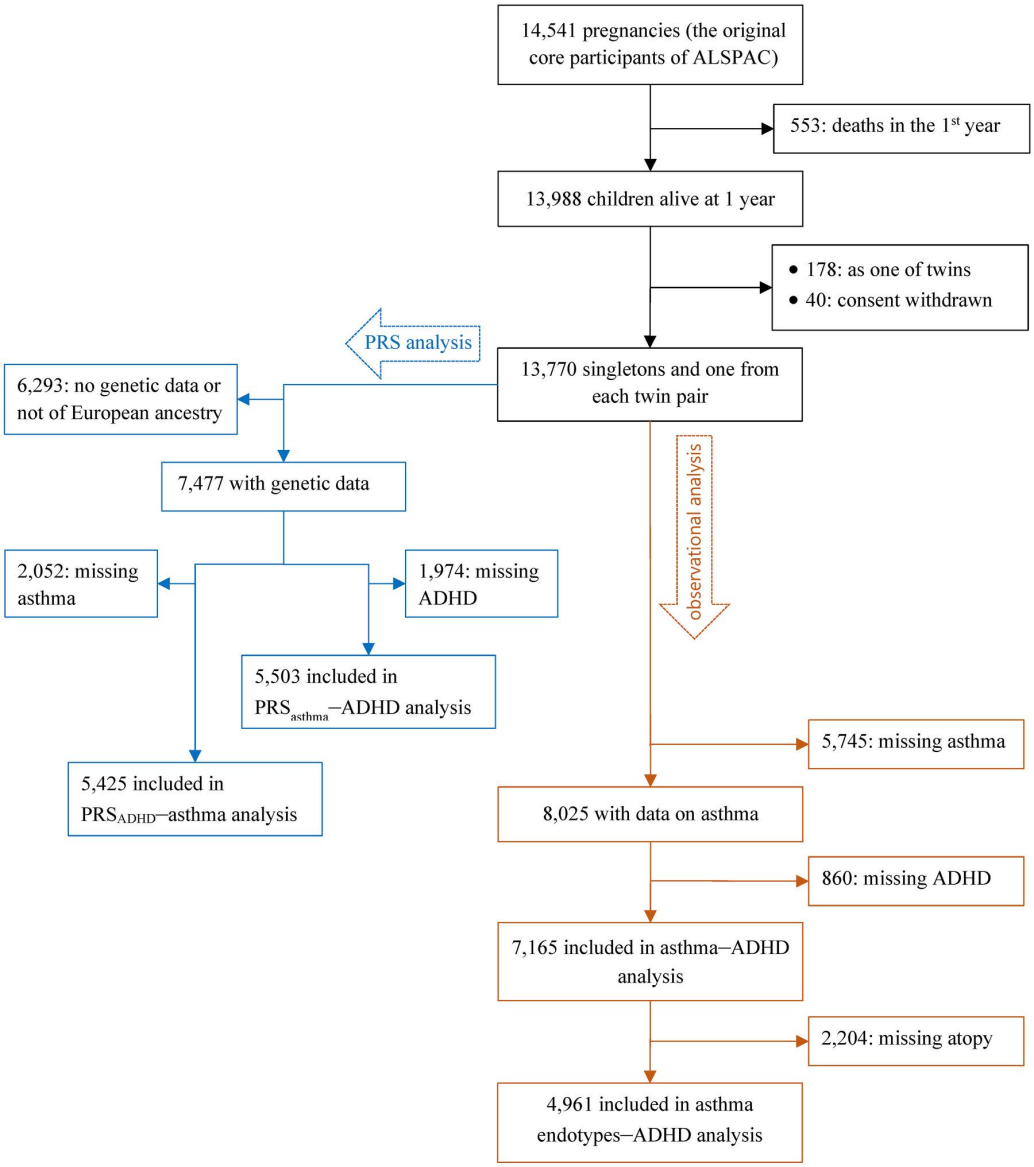
Flow diagram.

Figure 2 shows details on the sample sizes of various observational and PRS analyses. To deal with missing data for shared early-life risk factors, we used the missing-indicator method for categorical covariates and stochastic regression imputation for continuous covariates.

In our observational analyses, we first explored the impact of controlling for individual shared risk factors (potential confounders; listed in Table 1) on the association between asthma or asthma endotypes (exposure) and ADHD (outcome) in binary logistic regression models. We then controlled for these factors cumulatively to examine their combined effect on the asthma–ADHD association. We also carried out similar analyses by using asthma endotypes (four categories, with neither asthma nor atopy as the reference group). In *a priori* sensitivity analyses, we also tested the association of asthma with ADHD at 9 years.

**Table 1:** Characteristics of participants according to asthma and ADHD status at 7 years old.

	Total	Neither	ADHD only	Asthma only	ADHD and asthma	*P* value
*N* (%)	7165 (100)	5562 (77.6)	633 (8.8)	841 (11.7)	129 (1.8)	
Maternal age at delivery (years)	29.1 ± 4.5	29.2 ± 4.5	28.6 ± 4.7	29.0 ± 4.5	28.9 ± 5.0	.02
Maternal BMI (kg/m^2^)	22.9 ± 3.6	22.8 ± 3.5	22.6 ± 3.4	23.1 ± 3.9	23.9 ± 5.4	.06
Gestational age at birth (weeks)	39.5 ± 1.8	39.5 ± 1.8	39.3 ± 2.0	39.4 ± 1.8	39.3 ± 2.2	.03
Birth weight (g)	3433 ± 530	3447 ± 522	3336 ± 557	3421 ± 547	3386 ± 613	<.001
Free sugar intake at 32 weeks’ gestation (g/d)	58.2 ± 23.0	57.7 ± 22.5	59.9 ± 24.6	59.7 ± 24.4	62.7 ± 22.1	.002
Maternal energy intake at 32 weeks’ gestation (kJ/d)	7260 ± 1909	7256 ± 1891	7435 ± 2031	7161 ± 1922	7239 ± 1959	.09
Sex						
Female	3493 (48.8)	2874 (51.7)	214 (33.8)	368 (43.8)	37 (28.7)	<.001
Male	3672 (51.2)	2688 (48.3)	419 (66.2)	473 (56.2)	92 (71.3)	
Maternal education						
Certificate of Secondary Education	956 (13.3)	684 (12.3)	108 (17.1)	145 (17.2)	19 (14.7)	<.001
Vocational	604 (8.4)	451 (8.1)	67 (10.6)	69 (8.2)	17 (13.2)	
O-level	2468 (34.4)	1923 (34.6)	223 (35.2)	276 (32.8)	46 (35.7)	
A-level	1842 (25.7)	1463 (26.3)	146 (23.1)	209 (24.9)	24 (18.6)	
Degree	1155 (16.1)	946 (17.0)	73 (11.5)	120 (14.3)	16 (12.4)	
Missing	140 (2.0)	95 (1.7)	16 (2.5)	22 (2.6)	7 (5.4)	
Financial difficulty during pregnancy						
No	5998 (83.7)	4744 (85.3)	466 (73.6)	696 (82.8)	92 (71.3)	<.001
Yes	1123 (15.7)	787 (14.1)	161 (25.4)	140 (16.6)	35 (27.1)	
Missing	44 (0.6)	31 (0.6)	6 (0.9)	5 (0.6)	2 (1.6)	
Housing tenure at birth						
Mortgaged/owned	5803 (81.0)	4589 (82.5)	480 (75.8)	647 (76.9)	87 (67.4)	<.001
Council rented	565 (7.9)	387 (7.0)	64 (10.1)	92 (10.9)	22 (17.1)	
Non-council rented	454 (6.3)	329 (5.9)	52 (8.2)	58 (6.9)	15 (11.6)	
Unknown/missing	343 (4.8)	257 (4.6)	37 (5.8)	44 (5.2)	5 (3.9)	
Maternal smoking during pregnancy						
None	5691 (79.4)	4511 (81.1)	448 (70.8)	645 (76.7)	87 (67.4)	<.001
1–9/day	571 (8.0)	424 (7.6)	60 (9.5)	72 (8.6)	15 (11.6)	
10–19/day	616 (8.6)	423 (7.6)	86 (13.6)	88 (10.5)	19 (14.7)	
20+/day	271 (3.8)	192 (3.5)	38 (6.0)	34 (4.0)	7 (5.4)	
Missing	16 (0.2)	12 (0.2)	1 (0.2)	2 (0.2)	1 (0.8)	
Anxiety score in pregnancy						
0–3	2116 (29.5)	1741 (31.3)	137 (21.6)	222 (26.4)	16 (12.4)	<.001
4–6	2320 (32.4)	1835 (33.0)	188 (29.7)	263 (31.3)	34 (26.4)	
7–9	1436 (20.0)	1095 (19.7)	124 (19.6)	182 (21.6)	35 (27.1)	
10+	1066 (14.9)	726 (13.1)	162 (25.6)	140 (16.6)	38 (29.5)	
Missing	227 (3.2)	165 (3.0)	22 (3.5)	34 (4.0)	6 (4.7)	
Depression score in pregnancy						
0–3	2060 (28.8)	1682 (30.2)	129 (20.4)	231 (27.5)	18 (14.0)	<.001
4–6	2572 (35.9)	2051 (36.9)	197 (31.1)	281 (33.4)	43 (33.3)	
7–9	1544 (21.5)	1138 (20.5)	160 (25.3)	207 (24.6)	39 (30.2)	
10+	762 (10.6)	526 (9.5)	125 (19.7)	88 (10.5)	23 (17.8)	
Missing	227 (3.2)	165 (3.0)	22 (3.5)	34 (4.0)	6 (4.7)	
Any use of paracetamol during pregnancy						
No	2804 (39.1)	2284 (41.1)	212 (33.5)	269 (32.0)	39 (30.2)	<.001
Yes	3968 (55.4)	2988 (53.7)	383 (60.5)	520 (61.8)	77 (59.7)	
Missing	393 (5.5)	290 (5.2)	38 (6.0)	52 (6.2)	13 (10.1)	
Any use of antibiotics in pregnancy						
No	6078 (84.8)	4772 (85.8)	522 (82.5)	691 (82.2)	93 (72.1)	<.001
Yes	1087 (15.2)	790 (14.2)	111 (17.5)	150 (17.8)	36 (27.9)	
Pre-eclampsia						
No	6977 (97.4)	5424 (97.5)	615 (97.2)	815 (96.9)	123 (95.3)	.76
Yes	157 (2.2)	115 (2.1)	15 (2.4)	22 (2.6)	5 (3.9)	
Missing	31 (0.4)	23 (0.4)	3 (0.5)	4 (0.5)	1 (0.8)	

Categorical factors are presented as *n* (%) and continuous factors as mean ± SD.

BMI, body mass index.

In our genetic analyses, we first explored the association between estimated PRSs and the corresponding traits (asthma PRS–asthma and ADHD PRS–ADHD associations) by using binary or multinomial logistic regression models, adjusted for the first 10 principal components (PCs) of the ALSPAC genotype data to avoid population stratification bias [38]. To examine the shared genetics between asthma and ADHD, we tested asthma PRS–ADHD and ADHD PRS–asthma associations as per *z*-score changes, adjusted for sex and the first 10 PCs, across seven *P*-value thresholds.

In the MR analysis, we estimated causal effects by using the inverse-variance weighted (IVW) method [40]. IVW can be used to combine the causal effects of multiple genetic variants and it is equivalent to fitting a weighted linear regression of the gene–outcome associations on the gene–exposure associations, with the intercept term constrained to zero [41]. Therefore, IVW estimates assume that there are no pleiotropic effects or that pleiotropy is balanced and all genetic variants are valid instruments [40]. Using bidirectional two-sample MR, we tested the causal effects of genetic liability to asthma on ADHD and genetic liability to ADHD on asthma, investigating possible reverse causation. MR sensitivity analyses were carried out, including F-statistic, MR–Egger regression, weighted median estimator, leave-one-out analysis, and Cochran’s Q statistic.

We carried out statistical analyses by using Stata version 18.0 (StataCorp, TX, USA), PLINK v.1.9, and R version 4.2.2 (R Core Team, 2022) through R Studio (RStudio Team, 2022). We have provided further details in the supplementary material on asthma and ADHD definitions, covariates, PRS calculation, ALSPAC genetic data, MR analysis, and IV assumptions.

## Results

Table 1 shows the characteristics of participants included in the observational analyses. There were 970 (13.5%), 1193 (16.5%), 629 (8.8%), 1161 (21.4%), and 762 (10.6%) participants with asthma, eczema, hay fever, atopy, and ADHD, respectively.

### Shared early-life risk factors

In the crude model, we found a 35% higher risk of ADHD in children with asthma (OR 1.35, 95% confidence interval (CI) 1.10–1.65). Compared with participants without asthma or atopy, non-atopic asthma was associated with ADHD (OR 1.51, 95% CI 1.09–2.08), whereas atopic asthma was not (OR 1.00, 95% CI 0.69–1.45). We found no evidence of an association between ADHD and atopy (OR 0.87, 95% CI 0.70–1.09), eczema (OR 1.04, 95% CI 0.85–1.27), or hay fever (OR 1.06, 95% CI 0.81–1.37) in crude models.

**Figure 3:**
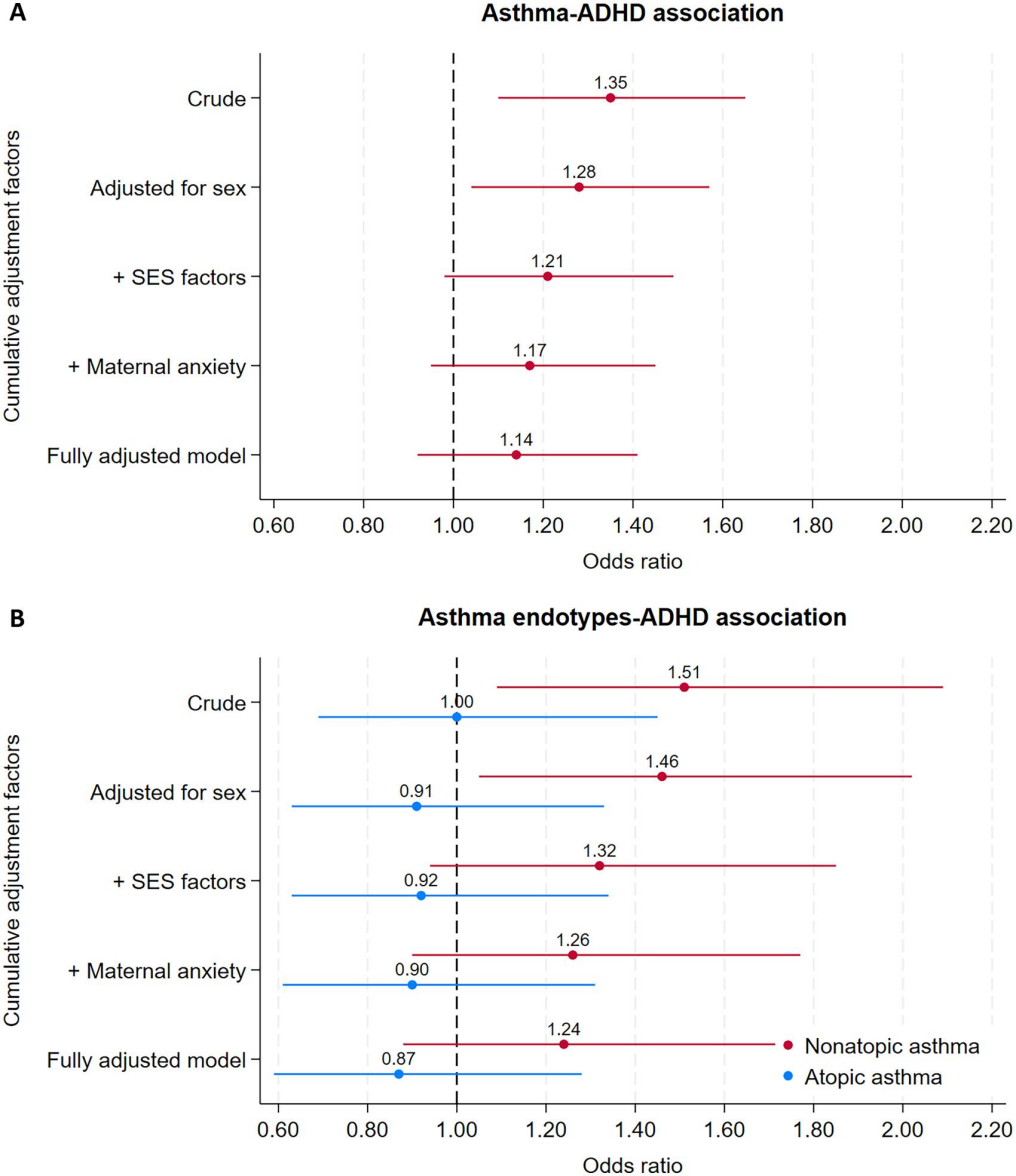
Odds ratios (95% CIs) for the association between (A) asthma or (B) its endotypes and ADHD after adjustment for early-life shared risk factors. (A) *N* = 7165; (B) *N* = 4961. The order is based on the extent of attenuation in individually adjusted models. The fully adjusted model included child sex, birth weight, gestational age at birth, and maternal factors, including age, SES factors (education, housing tenure at birth, financial difficulty, and index of multiple deprivation score), anxiety score, depression score, any use of paracetamol, smoking, free sugar intake, total energy intake, and antibiotic use, all in pregnancy, and BMI before pregnancy.

When we controlled the asthma–ADHD association for risk factors individually, almost all [except pre-eclampsia and body mass index (BMI)] attenuated the association to varying degrees; the strongest impact was made by the four socioeconomic status (SES) variables combined, followed by sex and maternal anxiety in pregnancy (Supplementary Table 4). In models cumulatively adjusted for early-life risk factors (Supplementary Table 5), the association substantially attenuated, particularly when adjusted for sex, SES factors, and maternal anxiety (OR 1.17, 95% CI 0.95–1.44). Further adjustment for the remaining risk factors only slightly reduced the association, such that, in the fully adjusted model, the OR (95% CI) was 1.14 (0.92–1.41) (Fig. 3 and Supplementary Table 5).

The non-atopic asthma–ADHD association was also attenuated by most of the individual risk factors, particularly the four SES factors, followed by maternal anxiety and smoking in pregnancy (Supplementary Table 4). After cumulative adjustment, the same pattern was seen, with substantial attenuation in the fully adjusted model (OR 1.24, 95% CI 0.88–1.74) (Supplementary Table 5). In the sensitivity analyses, the association between asthma at age 7 and ADHD at age 9 was slightly stronger than the association with ADHD at age 7, but the same pattern of attenuation was observed (Supplementary Table 6).

### Shared genetics

**Figure 4:**
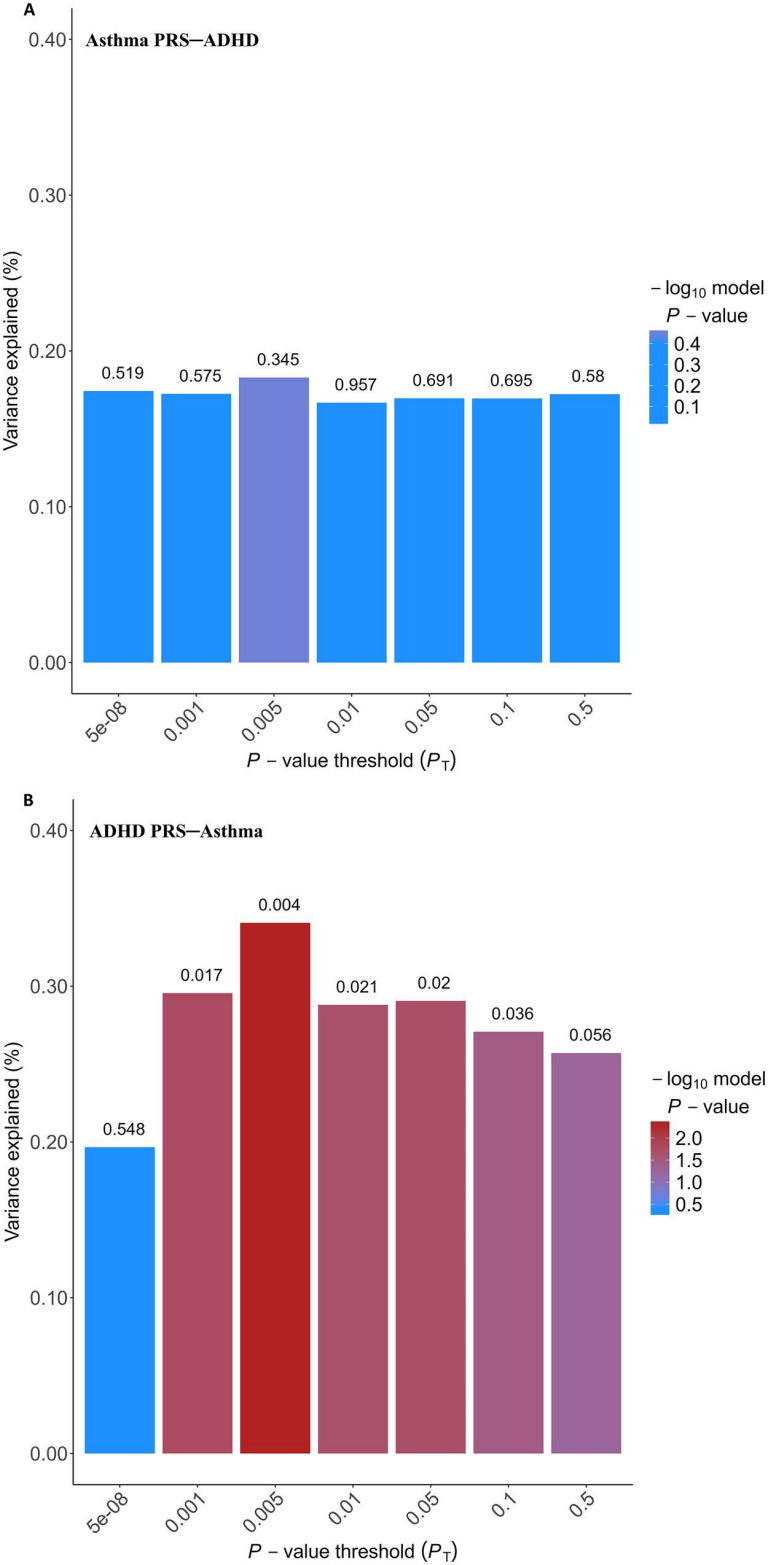
Associations of (A) PRSs for asthma with ADHD and (B) PRSs for ADHD with asthma at 7 years of age. *N* = 5503 (asthma PRSs–ADHD) and 5425 (ADHD PRSs–asthma). Associations were adjusted for the first 10 genetic PCs. Variance explained on the liability scale (%) based on Nagelkerke pseudo *R*^2^ in adjusted models.

We validated PRSs in ALSPAC participants; asthma PRSs were associated with asthma and its endotypes, atopic and non-atopic asthma, at all thresholds (Supplementary Tables 7 and 8) and ADHD PRSs were associated with ADHD at all thresholds except for 5 × 10^−8^ (Supplementary Table 9). Genetic liability to asthma was not associated with ADHD across all thresholds, whereas genetic liability to ADHD was associated with asthma at all thresholds except for 5 × 10^−8^ (Fig. 4 and Supplementary Table 10) and with non-atopic asthma at most thresholds (Table 2). In the sensitivity analysis, although the associations of ADHD PRSs were stronger with ADHD at age 9 (Supplementary Table 11) than ADHD at age 7  (suggesting stronger genetic influences), there was still little evidence of an association between asthma PRSs and ADHD at this later age across all thresholds (Supplementary Table 12).

**Table 2:** Association of PRS for ADHD with asthma endotypes at 7 years old.

	None	Atopy without asthma		Non-atopic asthma		Atopic asthma		Variance explained (%)^b^
		**RRR** ^a^ **(95% CI)**	*P* value	**RRR** ^a^ **(95% CI)**	*P* value	**RRR** ^a^ **(95% CI)**	*P* value	
*n* (%)	2963 (72.5)	596 (14.6)		260 (6.4)		270 (6.6)		
***P*-value thresholds for ADHD PRS**								
5 × 10^−1^	1.00	0.98 (0.89–1.07)	.59	1.12 (0.98–1.27)	.10	1.03 (0.90–1.16)	.69	0.86
1 × 10^−1^	1.00	0.97 (0.89–1.06)	.48	1.17 (1.02–1.32)	.02	1.02 (0.90–1.16)	.75	0.94
5 × 10^−2^	1.00	0.96 (0.88–1.05)	.42	1.17 (1.03–1.33)	.02	1.07 (0.95–1.22)	.27	0.98
1 × 10^−2^	1.00	0.93 (0.85–1.02)	.13	1.18 (1.04–1.34)	.01	1.06 (0.93–1.21)	.36	1.03
5 × 10^−3^	1.00	0.94 (0.86–1.03)	.18	1.15 (1.01–1.30)	.04	1.13 (0.99–1.28)	.06	1.04
1 × 10^−3^	1.00	0.93 (0.85–1.02)	.13	1.08 (0.95–1.23)	.22	1.12 (0.98–1.27)	.09	0.96
5 × 10^−8^	1.00	1.05 (0.96–1.15)	.26	1.01 (0.89–1.14)	.92	1.06 (0.93–1.20)	.37	0.83

*N* = 4089.

a Associations are presented as RRR per *z* score (95% CI), adjusted for sex and the first 10 genetic PCs.

b Variance explained on liability scale (%) based on Nagelkerke pseudo *R*^2^ in adjusted models.

RRR, relative-risk ratio.

Regarding other atopic phenotypes, we found inconsistent associations between eczema PRSs and ADHD (opposite directions at the highest and lowest thresholds). There was little evidence for an association between hay fever or allergic sensitization PRSs and ADHD (Supplementary Table 13). We also found little evidence for an association between ADHD PRSs and either eczema, hay fever, or atopy (Supplementary Table 14).

### Causal effect (two-sample MR)

The instrument strength estimated by using the F-statistic did not indicate weak instrument bias for asthma (31–188), eczema (30–117), hay fever (30–61), allergic sensitization (31–59), and ADHD (30–60) (variants in Supplementary Tables 15–19). All genetic variants used as proxies for asthma were available to be extracted from the ADHD GWAS, while only 21 (7 proxy variants) of the ADHD genetic variants were available in the asthma GWAS.

We found no evidence to support a causal effect of genetic liability to asthma on ADHD (Table 3). MR–Egger provided evidence of horizontal pleiotropic effects with a direction opposite to the IVW estimator, but with large CIs. No considerable heterogeneity was observed (Q = 31.1, *P *= .35) and the “leave-one-out” analysis did not identify any SNP as influential (Supplementary Table 20). There was weak evidence suggesting a causal effect of genetic liability to eczema and hay fever, but not allergic sensitization, on ADHD (Table 3), which were all confirmed in sensitivity analyses (Supplementary Tables 21–23).

**Table 3:** Bidirectional MR analysis of genetic liability to asthma, atopic dermatitis (eczema), atopic rhinitis (hay fever), and allergic sensitization on ADHD.

Exposure	Outcome	SNP count	**Causal effect estimates** ^a^		
			OR (95% CI)	*P* value	**Q *P*-value** ^b^
Asthma	ADHD	19	1.00 (0.94–1.06)	.89	.35
Eczema	ADHD	10	1.07 (1.00–1.15)	.05	.72
Hay fever	ADHD	3	1.12 (0.96–1.32)	.14	.23
Allergic sensitization	ADHD	11	1.00 (0.91–1.09)	.94	.01
ADHD	Asthma	20	1.06 (0.92–1.23)	.41	4.45 × 10^−04^
ADHD	Eczema	8	0.89 (0.76–1.03)	.11	.48
ADHD	Hay fever	8	0.91 (0.78–1.06)	.23	.82
ADHD	Allergic sensitization	8	0.91 (0.76–1.10)	.31	.88

a Using IVW estimator.

b *P* value for heterogeneity using Cochran’s Q statistic. A low *P* value indicates evidence of heterogeneity among the genetic instruments, which may reflect horizontal pleiotropy (violation of the MR assumption).

In the reverse direction, we found limited evidence to support a causal effect of genetic liability to ADHD on asthma (OR 1.06, 95% CI 0.92–1.23, *P* =.41). MR–Egger provided some evidence of horizontal pleiotropic effects with a direction opposite to the IVW estimator, but with large CIs. Causal effects estimated by using weighted median were comparable in direction to the IVW estimator. Evidence of heterogeneity was observed (Q = 46.3, *P* = 4.45 × 10^−4^), though, in the “leave-one-out” analysis, no genetic variant was identified as influential (Supplementary Table 20). There was little evidence for a causal effect of genetic liability to ADHD on eczema, hay fever, and allergic sensitization (Table 3), which were all confirmed in sensitivity analyses (Supplementary Tables 21–23).

## Discussion

Children with asthma, particularly non-atopic asthma, were at a higher risk of ADHD in crude models. Our results did not show evidence of a causal link between asthma and ADHD in either direction. We also did not find evidence of an association between genetic liability to asthma and ADHD; however, genetic liability to ADHD was associated with a higher risk of asthma. More importantly, early-life risk factors, particularly SES factors and anxiety in pregnancy, explained a substantial portion of the observed association between asthma and ADHD.

A few studies previously explored the causal link between asthma and ADHD [5–7]. In keeping with our findings, an MR analysis in UK Biobank did not support a causal effect of asthma on ADHD, although it showed evidence for a small positive causal effect of ADHD on asthma [5]. Consistent with our findings, two-sample MR studies also found little evidence of a causal effect of ADHD on asthma, but did not test the opposite direction [6, 7]. Limitations of MR analysis, such as instrument validity, population stratification, and low power, may still explain the lack of evidence supporting a causal link. Otherwise, the association found in observational studies will most likely be explained by residual confounding.

Confounding by shared genetics could be a potential explanation for the asthma–ADHD association and our findings support this, at least for genetic liability to ADHD. Of note, the discovery GWAS for asthma was smaller than the ADHD GWAS, and thus had relatively lower power. The only previous study that tested cross-trait PRS associations did not support a shared genetic background, but that study had a smaller sample size, and asthma was not doctor-diagnosed and was ascertained many years later, making it susceptible to recall bias [8]. Genetic overlap (seven shared loci) and a modest genome-wide genetic correlation (*R*_g_ = 0.197) between asthma and ADHD corroborated our findings [5]. However, shared genetics with ADHD was distinct for childhood- and adult-onset asthma, and genetic correlation supported the latter (*R*_g_ = 0.008 vs 0.280). The most recent GWAS for ADHD reported similar genetic correlations with asthma (*R*_g_ = 0.116–0.220) [42]. Co‐aggregation of asthma and ADHD in families was also explained mainly by genetic factors [43]. While only the PRS method assesses genetic liability to a trait at the individual level [38], these findings are complementary. The extent to which this genetic confounding can explain the observed association will be investigated in future studies.

We showed that the confounding effect of shared early-life risk factors can largely explain away the association between asthma and ADHD. Contrary to previous suggestions [28], ADHD was not associated with atopic asthma in this population, which fits with the lack of association with other atopic phenotypes (eczema and hay fever) and suggests that atopic immune mechanisms are unlikely to underlie the asthma–ADHD link. However, despite the much stronger association between non-atopic asthma and ADHD, it was also largely explained away by early-life risk factors.

Of the 23 studies included in Kass *et al.*’s meta-analysis [2], 7 did not control for any factor, while there was little agreement on which factors were controlled for in the remaining studies (Supplementary Table 24). Notably, the association was not controlled for any SES factor in 16 studies and, if controlled, often for no more than one or two factors. We have shown that rigorous adjustment for SES can explain away approximately one quarter of the asthma–ADHD effect estimate. SES is complex and multidimensional, thus inherently challenging to assess. Including multiple indicators of different components enables more rigorous control for their confounding effect. None of the four SES factors that we used in this study could individually attenuate the association as much as their collective impact.

These findings enhance our understanding of mechanisms underlying asthma–ADHD comorbidity. In particular, we identified maternal anxiety during pregnancy as a novel factor contributing to the asthma–ADHD association, independently of SES and other early-life risk factors. Unlike shared genetic susceptibility, most of these early-life risk factors are modifiable. Of course, any preventive potential hinges on the assumption that they are causally related to asthma and ADHD, which for example, in the case of maternal smoking and ADHD, may not be the case [23]. Instead, maternal genetic liability to ADHD was associated with multiple early-life exposures, including depression/anxiety symptoms and smoking during pregnancy [44]. It is therefore possible that the link between maternal anxiety and childhood ADHD is confounded by maternal genetic liability to ADHD. The link between maternal genetic liability to asthma and early-life risk factors has yet to be investigated.

### Strengths and limitations

The objective atopy measurements in ALSPAC enabled us to differentiate atopic from non-atopic asthma; previous studies have only examined overall asthma. Rich early-life data allowed us to probe more deeply than previous studies to explore the role of shared early-life risk factors, particularly SES and maternal anxiety. Previous studies identified childhood asthma through data collected in adulthood [5, 8]; our study was the first to use data collected in childhood.

The small sample size for the asthma endotypes resulted in wide 95% CIs and may be subject to type 2 error. Although SDQ is a valid instrument for identifying ADHD cases in population studies, we cannot rule out misclassifications, which may have attenuated the effect estimates. Our exploration of potential shared genetics was limited by the little variance in the phenotypes explained by common variants identified in GWASs—a common limitation of PRS studies despite improvements in recent years. Moreover, the ADHD PRS explained only a small proportion of variance in ADHD (Nagelkerke *R*^2^ < 1%), reflecting the polygenic nature of the disorder. We analysed a subset of ALSPAC participants with genetic data and longitudinal follow-up, which may have limited generalizability and led to underestimation of the total contribution of common variants to ADHD risk. In addition, some sample overlap between ALSPAC and the discovery GWAS for eczema and hay fever may have modestly inflated the PRS associations, so the effect estimates should be interpreted with caution, though the overall patterns remain informative. Assortative mating, population stratification, and selection biases in the GWASs can have confounding effects on MR findings [7] and yield biased estimates in PRS analysis [38]. An MR analysis using non-atopic asthma could be more informative, subject to the availability of GWASs for non-atopic asthma. The association between asthma and ADHD was shown to be phenotype-dependent, with a much stronger association between early-onset persistent than remittent phenotypes, as well as a distinct relationship between late-onset phenotypes [45], which might have implications for asthma medications, too. Our findings are not generalizable to all these sub-phenotypes and may not accurately reflect medication use. We did not correct *P* values for multiple testing in the genetic analyses because variants in PRSs at lower thresholds are a subset of variants in PRSs at higher thresholds. However, we cannot completely rule out chance findings, although the ADHD PRS at the 5 × 10^−3^ threshold still passes a Bonferroni-corrected multiple testing burden of *P *< .007 (.05/7), which is probably too conservative in this case.

## Conclusions

The asthma–ADHD association is unlikely to be causal but, in mid-childhood, it might partly reflect shared genetic liability and especially shared early-life risk factors. SES factors and maternal anxiety during pregnancy, in particular, may have a role in asthma–ADHD comorbidity.

## Ethics approval

Ethical approval for the study was obtained from the ALSPAC Ethics and Law Committee and the Local Research Ethics Committees. Informed consent for the use of data collected via questionnaires and clinics was obtained from participants, following the recommendations of the ALSPAC Ethics and Law Committee at the time. Consent for biological samples was collected in accordance with the Human Tissue Act (2004).

## Supplementary Material

dyag074_Supplementary_Data

## Data Availability

The informed consent obtained from ALSPAC participants does not allow the data to be made freely available through any third-party-maintained public repository. However, data used for this submission can be made available upon request to the ALSPAC Executive. The ALSPAC data-management plan describes in detail the policy regarding data sharing, which is through a system of managed open access. Full instructions for applying for data access can be found at http://www.bristol.ac.uk/alspac/researchers/access/. The ALSPAC study website contains details of all the data that are available (http://www.bristol.ac.uk/alspac/researchers/our-data/). The analysis codes are available at https://github.com/mtalaei/AstADHD.git.

## References

[1] Cortese S , Sun S, Zhang J et al Association between attention deficit hyperactivity disorder and asthma: a systematic review and meta-analysis and a Swedish population-based study. Lancet Psychiatry 2018;5:717–26. 10.1016/S2215-0366(18)30224-4 30054261

[2] Kaas TH , Vinding RK, Stokholm J, Bønnelykke K, Bisgaard H, Chawes BL. Association between childhood asthma and attention deficit hyperactivity or autism spectrum disorders: a systematic review with meta-analysis. Clin Exp Allergy 2021;51:228–52. 10.1111/cea.13750 32997856

[3] Schmitt J , Buske-Kirschbaum A, Roessner V. Is atopic disease a risk factor for attention-deficit/hyperactivity disorder? A systematic review. Allergy 2010;65:1506–24. 10.1111/j.1398-9995.2010.02449.x 20716320

[4] Davey Smith G , Hemani G. Mendelian randomization: genetic anchors for causal inference in epidemiological studies. Hum Mol Genet 2014;23:R89–98. 10.1093/hmg/ddu328 25064373 PMC4170722

[5] Zhu Z , Zhu X, Liu CL et al Shared genetics of asthma and mental health disorders: a large-scale genome-wide cross-trait analysis. Eur Respir J 2019;54:1901507. 10.1183/13993003.01507-2019 31619474

[6] Zhang X , Zhang R, Zhang Y, Lu T. Associations between attention-deficit/hyperactivity disorder and allergic diseases: a two-sample Mendelian randomization study. Front Psychiatry 2023;14:1185088. 10.3389/fpsyt.2023.1185088 37484661 PMC10356558

[7] Leppert B , Riglin L, Wootton RE et al The effect of attention deficit/hyperactivity disorder on physical health outcomes: a 2-sample mendelian randomization study. Am J Epidemiol 2021;190:1047–55. 10.1093/aje/kwaa273 33324987 PMC8168225

[8] Leffa DT , Horta B, Barros FC et al Association between Polygenic Risk Scores for ADHD and Asthma: a birth cohort investigation. J Atten Disord 2022;26:685–95. 10.1177/10870547211020111 34078169

[9] Holmberg K , Lundholm C, Anckarsater H, Larsson H, Almqvist C. Impact of asthma medication and familial factors on the association between childhood asthma and attention-deficit/hyperactivity disorder: a combined twin- and register-based study: Epidemiology of Allergic Disease. Clin Exp Allergy 2015;45:964–73. 10.1111/cea.12529 25772649

[10] Mogensen N , Larsson H, Lundholm C, Almqvist C. Association between childhood asthma and ADHD symptoms in adolescence—a prospective population-based twin study. Allergy 2011;66:1224–30. 10.1111/j.1398-9995.2011.02648.x 21599704

[11] Shaheen SO , Newson RB, Ring SM, Rose-Zerilli MJ, Holloway JW, Henderson AJ. Prenatal and infant acetaminophen exposure, antioxidant gene polymorphisms, and childhood asthma. J Allergy Clin Immunol 2010;126:1141–8 e7. 10.1016/j.jaci.2010.08.047 21051083 PMC4907348

[12] Ystrom E , Gustavson K, Brandlistuen RE et al Prenatal exposure to acetaminophen and risk of ADHD. Pediatrics 2017;140:e20163840. 10.1542/peds.2016-3840 PMC5654387 29084830

[13] Otten K , Keller L, Puiu AA et al Pre- and postnatal antibiotic exposure and risk of developing attention deficit hyperactivity disorder-A systematic review and meta-analysis combining evidence from human and animal studies. Neurosci Biobehav Rev 2022;140:104776. 10.1016/j.neubiorev.2022.104776 35842009

[14] Duong QA , Curtis N, Zimmermann P. The association between prenatal antibiotic exposure and adverse long-term health outcomes in children: a systematic review and meta-analysis. J Infect 2025;90:106377. 10.1016/j.jinf.2024.106377 39675435

[15] Andersson NW , Hansen MV, Larsen AD, Hougaard KS, Kolstad HA, Schlünssen V. Prenatal maternal stress and atopic diseases in the child: a systematic review of observational human studies. Allergy 2016;71:15–26. 10.1111/all.12762 26395995 PMC5054838

[16] Grizenko N , Fortier ME, Zadorozny C et al Maternal stress during pregnancy, ADHD symptomatology in children and genotype: gene-environment interaction. J Can Acad Child Adolesc Psychiatry 2012;21:9–15. 22299010 PMC3269259

[17] Bedard A , Northstone K, Henderson AJ, Shaheen SO. Maternal intake of sugar during pregnancy and childhood respiratory and atopic outcomes. Eur Respir J 2017;50:1700073. 10.1183/13993003.00073-2017 28679610 PMC5540678

[18] Choi CS , Kim P, Park JH et al High sucrose consumption during pregnancy induced ADHD-like behavioral phenotypes in mice offspring. J Nutr Biochem 2015;26:1520–6. 10.1016/j.jnutbio.2015.07.018 26452319

[19] Dachew BA , Scott JG, Mamun A, Alati R. Pre-eclampsia and the risk of attention-deficit/hyperactivity disorder in offspring: findings from the ALSPAC birth cohort study. Psychiatry Res 2019;272:392–7. 10.1016/j.psychres.2018.12.123 30605798

[20] Stokholm J , Sevelsted A, Anderson UD, Bisgaard H. Preeclampsia Associates with asthma, allergy, and eczema in childhood. Am J Respir Crit Care Med 2017;195:614–21. 10.1164/rccm.201604-0806OC 27626972

[21] Andersen CH , Thomsen PH, Nohr EA, Lemcke S. Maternal body mass index before pregnancy as a risk factor for ADHD and autism in children. Eur Child Adolesc Psychiatry 2018;27:139–48. 10.1007/s00787-017-1027-6 28712019

[22] Scholtens S , Wijga AH, Brunekreef B et al Maternal overweight before pregnancy and asthma in offspring followed for 8 years. Int J Obes (Lond) 2010;34:606–13. 10.1038/ijo.2009.194 19786965

[23] Thapar A , Rice F, Hay D et al Prenatal smoking might not cause attention-deficit/hyperactivity disorder: evidence from a novel design. Biol Psychiatry 2009;66:722–7. 10.1016/j.biopsych.2009.05.032 19596120 PMC2756407

[24] Leppert B , Havdahl A, Riglin L et al Association of maternal neurodevelopmental risk alleles with early-life exposures. JAMA Psychiatry 2019;76:834–42. 10.1001/jamapsychiatry.2019.0774 31042271 PMC6495368

[25] Miyazaki C , Koyama M, Ota E et al Allergic diseases in children with attention deficit hyperactivity disorder: a systematic review and meta-analysis. BMC Psychiatry 2017;17:120. 10.1186/s12888-017-1281-7 28359274 PMC5374627

[26] Schans JV , Cicek R, de Vries TW, Hak E, Hoekstra PJ. Association of atopic diseases and attention-deficit/hyperactivity disorder: a systematic review and meta-analyses. Neurosci Biobehav Rev 2017;74:139–48. 10.1016/j.neubiorev.2017.01.011 28111269

[27] Wang LJ , Yu YH, Fu ML et al Attention deficit-hyperactivity disorder is associated with allergic symptoms and low levels of hemoglobin and serotonin. Sci Rep 2018;8:10229. 10.1038/s41598-018-28702-5 29980754 PMC6035203

[28] Yang CF , Yang CC, Wang IJ. Association between allergic diseases, allergic sensitization and attention-deficit/hyperactivity disorder in children: a large-scale, population-based study. J Chin Med Assoc 2018;81:277–83. 10.1016/j.jcma.2017.07.016 29239851

[29] Boyd A , Golding J, Macleod J et al Cohort Profile: The 'children of the 90s’--the index offspring of the Avon Longitudinal Study of Parents and Children. Int J Epidemiol 2013;42:111–27. 10.1093/ije/dys064 22507743 PMC3600618

[30] Fraser A , Macdonald-Wallis C, Tilling K et al Cohort Profile: The Avon Longitudinal Study of Parents and Children: ALSPAC mothers cohort. Int J Epidemiol 2013;42:97–110. 10.1093/ije/dys066 22507742 PMC3600619

[31] Cornish RP , Henderson J, Boyd AW, Granell R, Van Staa T, Macleod J. Validating childhood asthma in an epidemiological study using linked electronic patient records. BMJ Open 2014;4:e005345. 10.1136/bmjopen-2014-005345 PMC4010849 24760357

[32] Roberts G , Peckitt C, Northstone K et al; Jul ALSPAC Study Team. Relationship between aeroallergen and food allergen sensitization in childhood. Clin Exp Allergy 2005;35:933–40. 10.1111/j.1365-2222.2005.02280.x 16008681

[33] Goodman R. The Strengths and Difficulties Questionnaire: a research note. J Child Psychol Psychiatry 1997;38:581–6. 10.1111/j.1469-7610.1997.tb01545.x 9255702

[34] Hall CL , Guo B, Valentine AZ et al The validity of the Strengths and Difficulties Questionnaire (SDQ) for children with ADHD symptoms. PLoS One 2019;14:e0218518. 10.1371/journal.pone.0218518 31216327 PMC6583960

[35] Algorta GP , Dodd AL, Stringaris A, Youngstrom EA. Diagnostic efficiency of the SDQ for parents to identify ADHD in the UK: a ROC analysis. Eur Child Adolesc Psychiatry 2016;25:949–57. 10.1007/s00787-015-0815-0 26762184 PMC4990620

[36] Textor J , van der Zander B, Gilthorpe MS, Liskiewicz M, Ellison GT. Robust causal inference using directed acyclic graphs: the R package ‘dagitty’. Int J Epidemiol 2016;45:1887–94. 10.1093/ije/dyw341 28089956

[37] Dapas M , Lee YL, Wentworth-Sheilds W, Im HK, Ober C, Schoettler N. Revealing polygenic pleiotropy using genetic risk scores for asthma. HGG Adv 2023;4:100233. 10.1016/j.xhgg.2023.100233 37663543 PMC10474095

[38] Choi SW , Mak TS, O’Reilly PF. Tutorial: a guide to performing polygenic risk score analyses. Nat Protoc 2020;15:2759–72. 10.1038/s41596-020-0353-1 32709988 PMC7612115

[39] Dudbridge F. Power and predictive accuracy of polygenic risk scores. PLoS Genet 2013;9:e1003348. 10.1371/journal.pgen.1003348 23555274 PMC3605113

[40] Burgess S , Davey Smith G, Davies NM et al Guidelines for performing Mendelian randomization investigations. Wellcome Open Res 2019;4:186. 10.12688/wellcomeopenres.15555.2 32760811 PMC7384151

[41] Pagoni P , Dimou NL, Murphy N, Stergiakouli E. Using Mendelian randomisation to assess causality in observational studies. Evid Based Ment Health 2019;22:67–71. 10.1136/ebmental-2019-300085 30979719 PMC10270458

[42] Demontis D , Walters GB, Athanasiadis G et al; Feb iPSYCH-Broad Consortium. Genome-wide analyses of ADHD identify 27 risk loci, refine the genetic architecture and implicate several cognitive domains. Nat Genet 2023;55:198–208. 10.1038/s41588-022-01285-8 36702997 PMC10914347

[43] Sun S , Kuja-Halkola R, Chang Z, Cortese S, Almqvist C, Larsson H. Familial liability to asthma and ADHD: a Swedish national register-based study. JCPP Adv 2021;1:e12044. 10.1002/jcv2.12044 37431403 PMC10242819

[44] Havdahl A , Wootton RE, Leppert B et al Associations between pregnancy-related predisposing factors for offspring neurodevelopmental conditions and parental genetic liability to attention-deficit/hyperactivity disorder, autism, and schizophrenia: The Norwegian Mother, Father and Child Cohort Study (MoBa). JAMA Psychiatry 2022;79:799–810. 10.1001/jamapsychiatry.2022.1728 35793100 PMC9260642

[45] Leffa DT , Caye A, Santos I et al Attention-deficit/hyperactivity disorder has a state-dependent association with asthma: the role of systemic inflammation in a population-based birth cohort followed from childhood to adulthood. Brain Behav Immun 2021;97:239–49. 10.1016/j.bbi.2021.08.004 34371132

