# Supplementary material for: Comorbidity of asthma and attention deficit hyperactivity disorder in childhood: causal, shared early-life risk factors, or shared genetic liability?
Source: Int J Epidemiol. 2026 May 23;55(3):dyag074. doi: 10.1093/ije/dyag074 (PMC13198363; doi:10.1093/ije/dyag074)
Supplement: dyag074_Supplementary_Data [file dyag074_supplementary_data.zip › 08-May-2026_051430_ije-2025-06-1129-File007.pdf]

International Journal of Epidemiology, 2026, dyag074
https://doi.org/10.1093/ije/dyag074

Online supplementary materials

Comorbidity of asthma and attention deficit hyperactivity disorder in childhood: causal, shared early-life risk factors, or shared genetic liability?

Mohammad Talaei, Panagiota Pagoni, Evie Stergiakouli, Seif O. Shaheen

Corresponding author:

Dr Mohammad Talaei, Wolfson Institute of Population Health, Queen Mary University of London, Charterhouse Square, London EC1M 6B, UK. E-mail: m.talaei@qmul.ac.uk; Tel: +44(0)20 7882 2499

Contents

Comorbidity of asthma and attention deficit hyperactivity disorder in childhood: causal, shared early-life risk factors, or shared genetic liability? **1**

Further details **4**

Asthma and atopy 4

Determining ADHD 4

Sample size 5

Early life risk factors 5

Imputation 6

Genetic data in ALSPAC 6

Mendelian Randomization 7

Instrument extraction 7

Harmonisation 7

Sensitivity analyses 7

References **10**

Supplementary tables and figures **12**

sFigure 1. Directed acyclic graph (DAG) for the association between asthma and attention deficit hyperactivity disorder (ADHD). 12

sFigure 2. Distribution of polygenic risk scores (PRS) at different *P*-value thresholds for asthma and attention deficit hyperactivity disorder (ADHD). 13

sTable 1. Information about genome-wide association studies used in calculation of Polygenic risk scores and in Mendelian randomization analyses. 14

sTable 2. Number of variants used to calculate polygenic risk scores (PRS) at each *P*-value threshold for asthma and attention deficit hyperactivity disorder (ADHD). 15

sTable 3. Correlation (r) of Polygenic Risk Scores (PRS) for asthma and attention deficit hyperactivity disorder (ADHD). 16

sTable 4. Odds ratio (95% confidence interval) for attention deficit hyperactivity disorder according to asthma status or asthma endotypes, all at 7 years, adjusted for each shared early life risk factor individually. 17

sTable 5. Odds ratio (95% confidence interval) for attention deficit hyperactivity disorder according to asthma status or asthma endotypes, all at 7 years, cumulatively adjusted for shared risk factors. 18

sTable 6. Odds ratio (95% confidence interval) for attention deficit hyperactivity disorder at 9 years according to asthma status or asthma endotypes at 7 years, cumulatively adjusted for shared risk factors 19

sTable 7. The association of polygenic risk scores for asthma with asthma at 7 years and indicators of goodness of fit. 20

sTable 8. The association of polygenic risk scores for asthma with asthma endotypes at 7 years and indicators of goodness of fit. 21

sTable 9. Association of polygenic risk scores for attention deficit hyperactivity disorder (ADHD) with ADHD at 7 years and indicators of goodness of fit. 22
sTable 10. Associations of polygenic risk scores for asthma with attention deficit hyperactivity disorder at 7 years and polygenic risk scores for attention deficit hyperactivity disorder with asthma at 7 years. 23
sTable 11. Association of polygenic risk scores for attention deficit hyperactivity disorder (ADHD) with ADHD at 9 years and indicators of goodness of fit. 24
sTable 12. Association of polygenic risk score for asthma with attention deficit hyperactivity disorder at 9 years. 25
sTable 13. Association of polygenic risk score for eczema and hay fever with attention deficit hyperactivity disorder at 7 years of age. 26
sTable 14. Association of polygenic risk score for attention deficit hyperactivity disorder with eczema and hay fever at 7 years of age. 27
sTable 15. Characteristics of 19 genetic variants associated with genetic liability to asthma. 28
sTable 16. Characteristics of 13 genetic variants associated with genetic liability to atopic dermatitis (eczema). 29
sTable 17. Characteristics of 3 genetic variants associated with genetic liability to Atopic rhinitis / Hay fever. 30
sTable 18. Characteristics of 11 genetic variants associated with genetic liability to allergic sensitisation (atopy). 31
sTable 19. Characteristics of 26 genetic variants associated with genetic liability to attention deficit hyperactivity disorder (ADHD). 32
sTable 20. Bidirectional causal effect estimates of genetic liability to asthma and ADHD as estimated by IVW, MR-Egger and weighted median estimator. 33
sTable 21. Bidirectional causal effect estimates of genetic liability to atopic dermatitis (eczema) and as estimated by IVW, MR-Egger and weighted median estimator. 34
sTable 22. Bidirectional causal effect estimates of genetic liability to atopic rhinitis (hay fever) and ADHD as estimated by IVW, MR-Egger and weighted median estimator. 35
sTable 23. Bidirectional causal effect estimates of genetic liability to allergic sensitisation and ADHD as estimated by IVW, MR-Egger and weighted median estimator. 36
sTable 24. Factors that the association between asthma and ADHD were controlled for in previous studies.[*] 37

Further details

Asthma and atopy

We defined current doctor-diagnosed asthma at ~7 years (91 months) of age if mothers responded positively to the question "Has a doctor ever actually said that your study child has asthma?" and to at least one of the questions which asked if the child had had wheezing, wheezing and whistling in the chest, asthma, or asthma medication in the last 12 months.

A skin prick allergy test was done at age 7 years. Atopy was defined as a positive reaction to Dermatophagoides pteronyssinus (house dust mite), cat, grass pollens in which almost all the sensitised subjects reacted to at least one (in addition to the other allergens tested: peanuts, mixed tree nuts and egg and one of three other panels: animal danders, foods or aeroallergens) (1). The positive reaction was defined as maximum diameter of any detectable weal ≥3mm after subtracting positive saline reactions from histamine and allergen weals and excluding children unreactive to 1% histamine (1). We categorised participants into four mutually exclusive endotypes: none, atopy-only, atopic asthma, and non-atopic asthma. Current eczema and hay fever in children at age 7 years were defined by a positive answer to the question "Has your child had any of the following in the past 12 months?" that included eczema and hay fever items.

Determining ADHD

The Strengths and Difficulties Questionnaire (SDQ) (2) is a behavioural screening tool known for its high reliability and validity in detecting psychiatric diagnoses (3). One component of the SDQ evaluates symptoms of combined hyperactive-impulsive and inattentive ADHD. This subscale comprises five items (three hyperactive-impulsive and two inattentive items): 'Restless, overactive. Cannot stay still for long' (hyperactive-impulsive), 'Constantly fidgeting or squirming' (hyperactive-impulsive), 'Easily distracted, concentration wanders' (inattentive), 'Thinks things out before acting' (hyperactive-impulsive, reverse coded) and 'Sees tasks through to the end. Good attention span' (inattentive, reverse coded). These items are rated on a scale of 'Not True' (0), 'Somewhat True' (1), and 'Certainly True' (2), and are then combined to create a summary score. Higher scores on this scale indicate more behavioral issues, ranging from 0 to 10. These items mainly reflect hyperactivity-impulsivity and inattention, as intended, and show minimal overlap with other factors evaluated by the SDQ (4). Mothers provided reports on their children's levels of hyperactivity-impulsivity and inattention at different child ages. We used data obtained at ~7 (81 m) and 9 (108 m) years of age.

The hyperactivity-inattention items of the SDQ are specifically sensitive to ADHD symptomatology, supporting its construct validity (5). The score had good discriminative value [area under the curve (AUC) = 0.84] using registry diagnoses, indicating that the scale differentiates reasonably well between those who do and do not receive a clinical ADHD diagnosis (6). It also had strong screening properties

(AUCs of 0.91) for the parental SDQ reports against DSM-based phenotypes (7), with good (0.81) to excellent (0.96) AUCs across sex and age groups, and fair (0.70) to good (0.88) accuracy for discriminating ADHD from other diagnoses (8). Goodman (2), who developed SDQ, suggested a cutoff score of ≥7 on the SDQ hyperactivity-inattention subscale to identify the top 10% of the population. It has been used by many studies (9-11) and was strongly associated with increased risk of later clinically diagnosed ADHD in a large population cohort (6).

Sample size

Of the 13,770 singleton children, or one from each twin pair alive at 1 year of age, 7,477 children of European ancestry had good quality genetic data resulting in 5,503 and 5,425 individuals included in the PRS analyses with ADHD and asthma outcomes, respectively. Information on asthma and ADHD was available for 7,165 children, of whom data were also available on atopy for 4,961. These were the sample sizes included in the observational analyses of asthma and asthma endotypes, respectively (see supplementary Figure S3).

Early life risk factors

These factors included child sex, birth weight (g), gestational age at birth (w), and maternal factors including age (y), education (5 categories), housing tenure at birth (3 categories), financial difficulty (binary), index of multiple deprivation score (quintiles), anxiety score in pregnancy (4 categories), depression score in pregnancy (4 categories), any use of paracetamol in pregnancy (binary), smoking (4 categories), and free sugar intake (g/d), and body mass index (BMI) (Kg/m^2) before pregnancy (continuous).

The Index of Multiple Deprivation (IMD) is a composite area-level indicator provided by the UK government, derived from seven domains: income, employment, education, health, crime, housing, and living environment (12, 13). The living location was defined using the 2001 Census urban/rural indicator by postcode, and data on the IMD were obtained via postcode linkage (14). Data on maternal ethnicity and other indicators of socioeconomic status (maternal education, housing tenure and financial difficulty in pregnancy) were collected via questionnaire at various time points during pregnancy (8, 18, and 32 weeks of gestation) and at 8 weeks postpartum. Educational attainment was categorised using UK-specific qualifications, which are mapped to internationally comparable levels as follows: Certificate of Secondary Education (lower secondary education), Vocational (upper secondary vocational education); O-level (upper secondary education), A-level (post-secondary/pre-university education), and university degree (tertiary education). Maternal anxiety and depression were measured using the corresponding subscales of the Crown-Crisp Experiential Index (CCEI), a validated self-rating inventory (15) that results in scores ranging from 0 to 16. CCEI was administered to women during pregnancy as part of a self-completion questionnaire at 18 and 32 weeks of gestation (16); we used the maximum reported

score. Mothers were also asked whether they had used paracetamol in the previous 3 months at 18 and 32 weeks of pregnancy; we considered any use throughout pregnancy. Information on antibiotic use was derived from data collected from mothers at 8, 18, and 32 weeks of pregnancy (any antibiotic use during pregnancy). Mothers were asked about their weight before pregnancy, and their height was measured, both at 12 weeks of gestation. Pre-pregnancy BMI was calculated as weight (kg) divided by height squared (m^2).

Imputation

To deal with missing data in covariates (shared early life risk factors), we used the missing-indicator method for categorical covariates (i.e., adding a missing category) and stochastic regression imputation for continuous covariates (16). In stochastic regression imputation, the missing data point is substituted with a predicted value derived from a regression model plus a residual factor that accounts for the uncertainty associated with the prediction. We first built prediction models exploring all biologically plausible factors available that could theoretically predict our numerical covariates with missing values, namely maternal BMI, energy intake, sugar intake, and birth weight and used items that maximised R^2 for the prediction model. For example, the optimum model for maternal BMI included age (year), IMD, parity, total caloric intake (kJ/d), leisure-time physical activity (weighted index score), and PRS_{BMI} as calculated before (17). For the stochastic regression imputation, we used the 'mi impute naivereg' command in Stata 18 with two iterations and then used the average estimated values. In cases where data were missing for some components of the prediction models, we used smaller models that excluded those factors for those particular participants. The number (%) of participants with imputed values included in our observational analysis was 582 (8.1%) for maternal BMI, 305 (4.3%) for total caloric and sugar intake, and 84 (1.2%) for birth weight.

Genetic data in ALSPAC

A total of 10,015 mothers in ALSPAC were genotyped on the Illumina Human660W quad genome-wide single nucleotide polymorphism (SNP) genotyping platform at the Center National Genotypage, and genotypes were identified using Illumina GenomeStudio. A total of 9,912 ALSPAC children were genotyped on the Illumina HumanHap550 quad chip genotyping platforms by 23andme subcontracting the Wellcome Trust Sanger Institute, Cambridge, UK, and the Laboratory Corporation of America, Burlington, NC, United States.

Quality control filtering was performed using PLINK v1.07 (18). Participants were excluded based on the following filters: (1) gender mismatches; (2) undetermined X chromosome heterozygosity; (3) over 3% missingness (children); over 5% missingness (mothers); (4) evidence of crypted relatedness (>10% of shared alleles identical by descent in children and >12.5% of shared alleles identical by descent in mothers); (5) non-European ancestry, assessed by multidimensional scaling analysis compared with

HapMap 2 individuals. SNPs were excluded based on the following filters: (1) minor allele frequency < 1%; (2) call rate < 95%, (3) Hardy–Weinberg equilibrium (HWE) $P < 5\times10^{-07}$. Maternal and offspring genotype data were combined and imputed using Impute v.2.2.2 against 1000 Genomes reference panel (v.1, phase 3, December 2013 release).

After performing quality control and excluding participants due to withdrawal of consent, genetic data were available for 7,921 mothers and 7,977 children of European ancestry. Consent for biological samples was collected in accordance with the Human Tissue Act (2004).

Mendelian Randomization

The ADHD GWAS consisted of Danish iPSYCH cohort (25,895 cases; 37,148 controls) from Denmark, the Icelandic deCODE cohort (8,281 cases; 137,993 controls) and the Psychiatric Genomics Consortium (PGC; 4,515 cases; 11,702 controls). Of the 66 studies used in the asthma GWAS, the only shared study was deCODE, but it made 6.9% of cases of the asthma GWAS.

Instrument extraction

Approximately independent genetic variants were identified ($r^2 < 0.01$ within a 10,000 kb window, $P < 5 \times 10^{-08}$) for each exposure of interest and corresponding log odds ratios and standard errors were extracted from summary level datasets. Genetic variants used as proxies for exposures were then extracted from the outcome GWAS. When a genetic variant was not present in the outcome GWAS, we identified proxy variants using the LDLink online tool (19) ($r^2 > 0.90$).

Harmonisation

Effect estimates for genetic variants-exposure association were coded to express the estimated effect per increasing allele, and corresponding genetic variants-outcome effect estimates were harmonised to match the alleles of exposure (ensuring associations are expressed per the same allele). We excluded ambiguous SNPs (i.e., SNPs with complementary alleles A/T or C/G where strand direction was unknown), as alignment of alleles between exposure and outcome was not always possible.

Sensitivity analyses

Because the validity of estimated causal effects depends on whether the MR assumptions hold, we assessed their validity through a series of sensitivity analyses. First, to test the strength of the genetic instruments, we used the F-statistic to assess weak-instrument bias (20). The F-statistic is a function of the variance explained by a set of genetic variants (R2), the number of genetic variants used and the sample size. An F-statistic < 10 provides evidence of weak instrument bias, and causal effect estimates are likely to be influenced. Secondly, we compared the causal effects estimated using IVW with those obtained using the MR-Egger regression and the weighted median estimator (21, 22). MR-Egger regression includes an unconstrained intercept term, in contrast to IVW, where the intercept is zero, and

therefore, the intercept term is a formal test for presence of horizontal pleiotropy. Moreover, the MR-Egger slope provides an estimate of the causal effect, adjusting for pleiotropy. The weighted median estimator equals the median of the weighted-ratio estimates of genetic variants and provides an unbiased causal-effect estimate when up to 50% of the genetic variants included in the analysis are invalid instruments. Consistent directionality of estimated causal effects across IVW, MR-Egger and weighted median indicates a true causal effect. Thirdly, we used leave-one-out analysis to explore the influence of each genetic variant on the estimated causal effect by systematically removing each variant and re-estimating the causal effects (23). Lastly, we used the Cochran's Q statistic to assess whether the effects of genetic variants were comparable (23, 24), and observed that substantial heterogeneity could be considered as an indication of invalid instruments.

Asthma

For genetic liability of asthma on ADHD, MR-Egger provided evidence of horizontal pleiotropic effects (OR = 1.02, 95% CI: 1.00–1.04, *P*-value = 0.09), and the directionality of estimated causal effect was opposite to the IVW estimator (OR = 0.84, 95% CI: 0.69–1.03, *P*-value = 0.10), but with large confidence intervals due to the limited number of genetic variants included in the analysis (**Table S19**). No considerable heterogeneity was observed between the genetic variants (Q = 19.71, *P*-value = 0.35), and leave-one-out analysis did not identify any SNP as influential.

In the reverse direction, MR-Egger provided some evidence of horizontal pleiotropic effect (OR = 1.08, 95% CI: 0.99–1.19, *P*-value = 0.07), and the directionality of the estimated causal effect was opposite to the IVW estimator (OR = 0.39, 95% CI: 0.14–1.13, *P*-value = 0.08), but with large confidence intervals due to the limited number of genetic variants included in the analysis. Causal effects estimated using the weighted median were comparable in direction to the IVW estimator. No considerable heterogeneity was observed between the genetic variants (Q = 5.64, *P*-value = 0.22) and in leave-one-out analysis we identified rs17531412 and rs1427829 as influential.

Eczema

For the causal effect of genetic liability to eczema on ADHD, MR-Egger and weighted median estimators produced directionally comparable results to the IVW estimator (**Table S20**). Moreover, no indication of horizontal pleiotropy was identified by the MR-Egger intercept term (OR =0.99, 95% CI: 0.95–1.03, *P*-value = 0.48). No considerable heterogeneity was observed among the genetic variants (Q = 6.18, *P*-value = 0.72), and leave-one-out analyses did not identify any SNP as influential.

In the reverse direction, the MR-Egger and weighted median estimators produced results that were directionally comparable to those of the IVW estimator. In addition, no indication of horizontal pleiotropy was identified by the MR-Egger intercept term (OR = 1.03, 95% CI: 0.96–1.09, *P*-value = 0.41). No considerable heterogeneity was observed among the genetic variants (Q = 6.52, *P*-value = 0.48) and leave-one-out analyses did not identify any SNP as influential.

Hay fever

For the causal effect of genetic liability to hay fever on ADHD, the weighted median estimator provided evidence of a causal effect of genetic liability to hay fever on ADHD (OR = 1.18, 95% CI: 1.01–1.38, *P*-value = 0.04) (**Table S21**). MR-Egger produced directionally comparable results to the IVW estimator. In addition, no indication of horizontal pleiotropy was identified by the MR-Egger intercept term (OR = 0.95, 95% CI: 0.86–1.04, *P*-value =0.28). No considerable heterogeneity was observed between the genetic variants (Q = 2.94, *P*-value = 0.23), and leave-one-out analyses did not identify any SNP as influential.

In the reverse direction, MR-Egger and weighted median estimators produced directionally comparable results to the IVW estimator. In addition, no indication of horizontal pleiotropy was identified by the MR-Egger intercept term (OR = 1.00, 95% CI: 0.94–1.06, *P*-value <0.99). No considerable heterogeneity was observed among the genetic variants (Q = 3.55, *P*-value = 0.82), and leave-one-out analyses did not identify any SNP as influential.

Allergic sensitisation

For the causal effect of genetic liability to allergic sensitisation on ADHD, MR-Egger and weighted median estimators produced directionally comparable results to the IVW estimator (**Table S22**). No indication of horizontal pleiotropy was identified by the MR-Egger intercept term (OR = 1.01, 95% CI: 0.94–1.08, *P*-value =0.82). There was some evidence of heterogeneity observed among the genetic variants (Q = 23.02, *P*-value = 0.01), but no SNPs were identified as influential in leave-one-out analyses.

In the reverse direction, MR-Egger and weighted median estimators produced directionally comparable results to the IVW estimator. No indication of horizontal pleiotropy was identified by the MR-Egger intercept term (OR = 0.96, 95% CI: 0.89–1.04, *P*-value =0.30). There was no evidence of heterogeneity observed between the genetic variants (Q = 3.03, *P*-value = 0.88), and no SNPs were identified as influential in leave-one-out analyses.

References

1. Roberts G, Peckitt C, Northstone K, Strachan D, Lack G, Henderson J, et al. Relationship between aeroallergen and food allergen sensitisation in childhood. Clin Exp Allergy. 2005;35(7):933–40.

2. Goodman R. The Strengths and Difficulties Questionnaire: a research note. J Child Psychol Psychiatry. 1997;38(5):581–6.

3. Goodman R, Ford T, Simmons H, Gatward R, Meltzer H. Using the Strengths and Difficulties Questionnaire (SDQ) to screen for child psychiatric disorders in a community sample. Br J Psychiatry. 2000;177:534–9.

4. Goodman R. Psychometric properties of the strengths and difficulties questionnaire. J Am Acad Child Adolesc Psychiatry. 2001;40(11):1337–45.

5. Grasso M, Lazzaro G, Demaria F, Menghini D, Vicari S. The Strengths and Difficulties Questionnaire as a Valuable Screening Tool for Identifying Core Symptoms and Behavioural and Emotional Problems in Children with Neuropsychiatric Disorders. Int J Environ Res Public Health. 2022;19(13).

6. Overgaard KR, Madsen KB, Oerbeck B, Friis S, Obel C. The predictive validity of the Strengths and Difficulties Questionnaire for child attention-deficit/hyperactivity disorder. Eur Child Adolesc Psychiatry. 2019;28(5):625–33.

7. Ullebo AK, Posserud MB, Heiervang E, Gillberg C, Obel C. Screening for the attention deficit hyperactivity disorder phenotype using the strength and difficulties questionnaire. Eur Child Adolesc Psychiatry. 2011;20(9):451–8.

8. Algorta GP, Dodd AL, Stringaris A, Youngstrom EA. Diagnostic efficiency of the SDQ for parents to identify ADHD in the UK: a ROC analysis. Eur Child Adolesc Psychiatry. 2016;25(9):949–57.

9. Korologou-Linden R, Anderson EL, Jones HJ, Davey Smith G, Howe LD, Stergiakouli E. Polygenic risk scores for Alzheimer's disease, and academic achievement, cognitive and behavioural measures in children from the general population. Int J Epidemiol. 2019;48(6):1972–80.

10. Huss M, Hölling H, Kurth B-M, Schlack R. How often are German children and adolescents diagnosed with ADHD? Prevalence based on the judgment of health care professionals: results of the German health and examination survey (KiGGS). European Child & Adolescent Psychiatry. 2008;17(1):52–8.

11. Stergiakouli E, Thapar A, Davey Smith G. Association of Acetaminophen Use During Pregnancy With Behavioral Problems in Childhood: Evidence Against Confounding. JAMA Pediatr. 2016;170(10):964–70.

12. Noble M, Wright G, Smith G, Dibben C. Measuring Multiple Deprivation at the Small-Area Level. Environment and Planning A: Economy and Space. 2006;38(1):169–85.

13. Department for Communities and Local Government. The English Index of Multiple Deprivation (IMD) 2015: Guidance 2015. Available from: https://www.gov.uk/government/statistics/english-indices-of-deprivation-2015.

14. Mummé M, Boyd A, Teyhan A, Thomas R, Timpson N. Characterising the index mothers in the Avon Longitudinal Study of Parents and Children (ALSPAC) who are also UKBioBank participants [version 1; peer review: 1 approved, 1 approved with reservations]. Wellcome Open Research. 2022;7(190).

15. Birtchnell J, Evans C, Kennard J. The total score of the Crown-Crisp Experiential Index: a useful and valid measure of psychoneurotic pathology. Br J Med Psychol. 1988;61(3):255–66.

16. Baraldi AN, Enders CK. An introduction to modern missing data analyses. J Sch Psychol. 2010;48(1):5–37.

17. Bond TA, Richmond RC, Karhunen V, Cuellar-Partida G, Borges MC, Zuber V, et al. Exploring the causal effect of maternal pregnancy adiposity on offspring adiposity: Mendelian randomisation using polygenic risk scores. BMC Med. 2022;20(1):34.

18. Stergiakouli E, Gaillard R, Tavare JM, Balthasar N, Loos RJ, Taal HR, et al. Genome-wide association study of height-adjusted BMI in childhood identifies functional variant in ADCY3. Obesity (Silver Spring). 2014;22(10):2252–9.

19. Machiela MJ, Chanock SJ. LDlink: a web-based application for exploring population-specific haplotype structure and linking correlated alleles of possible functional variants. Bioinformatics. 2015;31(21):3555–7.

20. Burgess S, Thompson SG, Collaboration CCG. Avoiding bias from weak instruments in Mendelian randomisation studies. Int J Epidemiol. 2011;40(3):755–64.

21. Bowden J, Davey Smith G, Burgess S. Mendelian randomisation with invalid instruments: effect estimation and bias detection through Egger regression. Int J Epidemiol. 2015;44(2):512–25.

22. Bowden J, Davey Smith G, Haycock PC, Burgess S. Consistent Estimation in Mendelian Randomization with Some Invalid Instruments Using a Weighted Median Estimator. Genet Epidemiol. 2016;40(4):304–14.

23. Burgess S, Thompson SG. Interpreting findings from Mendelian randomisation using the MR-Egger method. Eur J Epidemiol. 2017;32(5):377–89.

24. Pagoni P, Dimou NL, Murphy N, Stergiakouli E. Using Mendelian randomisation to assess causality in observational studies. Evid Based Ment Health. 2019;22(2):67–71.

Supplementary tables and figures

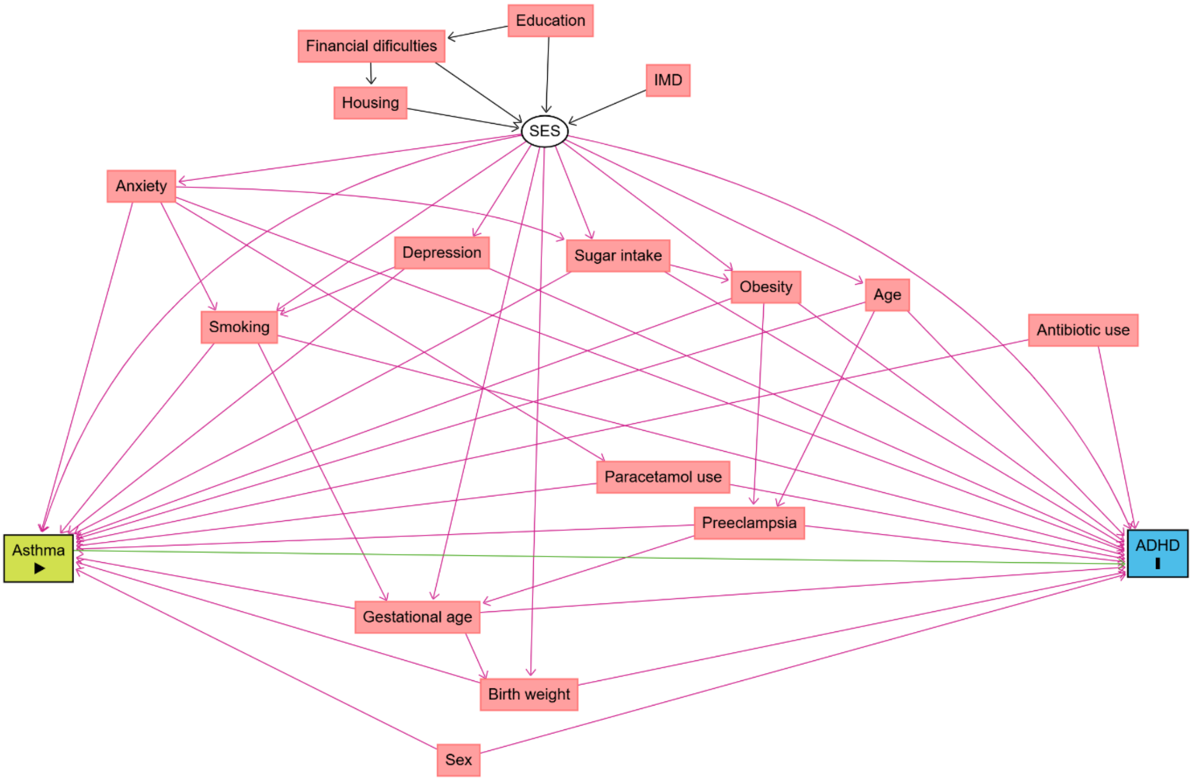

sFigure 1. Directed acyclic graph (DAG) for the association between asthma and attention-deficit hyperactivity disorder (ADHD).

Notes:

1. The direction between asthma and ADHD is for practical reasons; otherwise, we consider both directions plausible.
2. To make the DAG comprehensible, we simplified indicators of socioeconomic status (SES) into a latent factor to present an aggregated hypothetical effect on asthma, ADHD, and prenatal risk factors. Factors above and below the green line are prenatal and perinatal, respectively.

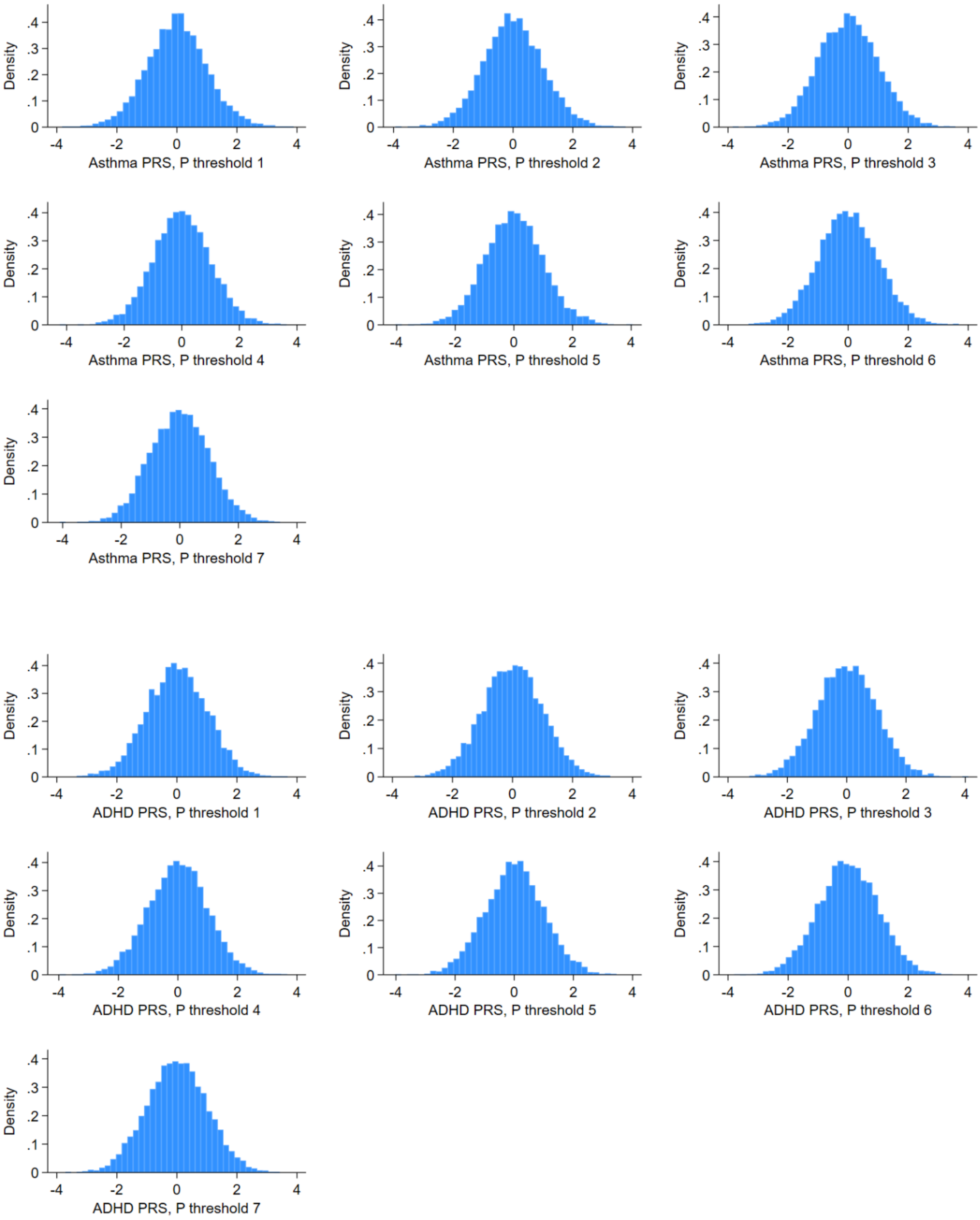

sFigure 2. Distribution of polygenic risk scores (PRS) at different *P*-value thresholds for asthma and attention deficit hyperactivity disorder (ADHD).

sTable 1. Information about genome-wide association studies used in calculation of polygenic risk scores and in Mendelian randomisation analyses.

Trait	PMID	First author	Year	N[*]	$N_{cases}/N_{controls}$
ADHD	36702997	Demontis D.	2023	225,534	38,691/186,843
Asthma	29273806	Demenais F	2018	142,486	23,948/118,538
Atopic dermatitis (eczema)	26482879	Paternoster L.	2015	116,863[†]	21,399/95,464
Allergic rhinitis (hay fever)	30013184	Waage J.	2018	212,120[†]	59,762/152,358
Allergic sensitization[§]	23817571	Bønnelykke K.	2013	15,854	5,798/10,056

[*] Sample includes children and adults in all studies

[†] GWAS includes ALSPAC samples.

[§] Asthma and/or Hay fever and/or eczema

sTable 2. Number of variants used to calculate polygenic risk scores (PRS) at each *P*-value threshold for asthma and attention deficit hyperactivity disorder (ADHD).

***P*-value thresholds**	5×10^{-1}	1×10^{-1}	5×10^{-2}	1×10^{-2}	5×10^{-3}	1×10^{-3}	5×10^{-8}
**Asthma**	129,239	40,650	23,884	6,864	3,967	1,223	35
**Atopic dermatitis/ eczema**	246,419	74,054	42,174	11,040	6,129	1,663	21
**Allergic rhinitis/ Hay fever**	244,499	71,099	39,830	10,060	5,489	1,290	3
**Allergic Sensitisation**	243,125	69,638	38,842	9,824	5,433	1,339	13
**ADHD**	199,893	64,686	38,457	11,535	6,830	2,181	13

sTable 3. Correlation (r) of Polygenic Risk Scores (PRS) for asthma and attention deficit hyperactivity disorder (ADHD).

***P*-value thresholds**	5×10^{-1}	1×10^{-1}	5×10^{-2}	1×10^{-2}	5×10^{-3}	1×10^{-3}	5×10^{-8}
**Asthma**							
5×10^{-1}	1						
1×10^{-1}	0.911	1					
5×10^{-2}	0.846	0.932	1				
1×10^{-2}	0.654	0.731	0.785	1			
5×10^{-3}	0.569	0.641	0.688	0.882	1		
1×10^{-3}	0.396	0.462	0.499	0.654	0.749	1	
5×10^{-8}	0.143	0.188	0.212	0.315	0.370	0.517	1
**ADHD**							
5×10^{-1}	1						
1×10^{-1}	0.927	1					
5×10^{-2}	0.870	0.943	1				
1×10^{-2}	0.716	0.783	0.836	1			
5×10^{-3}	0.651	0.716	0.764	0.912	1		
1×10^{-3}	0.501	0.555	0.598	0.725	0.793	1	
5×10^{-8}	0.132	0.151	0.166	0.218	0.253	0.324	1
**Cross-trait PRSs**							
Asthma-ADHD	0.038	0.041	0.046	0.041	0.034	0.002	-0.016

sTable 4. Odds ratio (95% confidence interval) for attention deficit hyperactivity disorder according to asthma status or asthma endotypes, all at 7 years, adjusted for each shared early life risk factor individually.

	Asthma*		Asthma endotypes†			
	**No**	**Yes**	**None**	**Atopy without asthma**	**Nonatopic asthma**	**Atopic asthma**
**ADHD** Cases/non-cases	633/5562	129/841	359/3204	63/666	48/284	34/303
Crude	1.00	1.35 (1.10-1.65)	1.00	0.84 (0.64-1.12)	1.51 (1.09-2.09)	1.00 (0.69-1.45)
**Adjusted for**						
Sex	1.00	1.28 (1.04-1.57)	1.00	0.78 (0.59-1.04)	1.46 (1.05-2.02)	0.91 (0.63-1.33)
SES						
Maternal education	1.00	1.31 (1.07-1.61)	1.00	0.85 (0.64-1.13)	1.44 (1.04-2.00)	1.00 (0.69-1.45)
IMD	1.00	1.33 (1.09-1.63)	1.00	0.84 (0.64-1.12)	1.50 (1.08-2.08)	1.00 (0.69-1.45)
Housing tenure at birth	1.00	1.31 (1.07-1.60)	1.00	0.85 (0.64-1.12)	1.43 (1.03-1.99)	0.99 (0.68-1.44)
Financial difficulty in pregnancy	1.00	1.32 (1.08-1.62)	1.00	0.87 (0.66-1.15)	1.44 (1.03-1.99)	1.00 (0.69-1.46)
All 4 SES factors	1.00	1.27 (1.04-1.57)	1.00	0.88 (0.66-1.16)	1.38 (0.99-1.92)	1.00 (0.69-1.45)
Anxiety score in pregnancy	1.00	1.28 (1.04-1.57)	1.00	0.86 (0.65-1.14)	1.40 (1.01-1.94)	0.97 (0.67-1.41)
Birth weight	1.00	1.34 (1.09-1.64)	1.00	0.86 (0.65-1.13)	1.47 (1.06-2.03)	1.01 (0.69-1.46)
Free sugar & total energy intake	1.00	1.34 (1.10-1.65)	1.00	0.84 (0.63-1.11)	1.51 (1.09-2.09)	0.99 (0.68-1.43)
Preeclampsia	1.00	1.35 (1.10-1.65)	1.00	0.85 (0.64-1.12)	1.50 (1.09-2.08)	1.00 (0.69-1.45)
Depression score in pregnancy	1.00	1.30 (1.06-1.60)	1.00	0.86 (0.65-1.14)	1.44 (1.04-2.00)	0.99 (0.68-1.43)
Gestational age	1.00	1.34 (1.10-1.64)	1.00	0.86 (0.65-1.13)	1.48 (1.07-2.05)	1.00 (0.69-1.46)
Paracetamol use in pregnancy	1.00	1.32 (1.07-1.61)	1.00	0.85 (0.64-1.13)	1.47 (1.06-2.04)	0.99 (0.69-1.44)
Maternal age	1.00	1.34 (1.09-1.64)	1.00	0.85 (0.64-1.13)	1.49 (1.08-2.07)	1.00 (0.69-1.45)
Smoking in pregnancy	1.00	1.31 (1.07-1.61)	1.00	0.87 (0.66-1.15)	1.42 (1.03-1.98)	1.00 (0.69-1.45)
BMI before pregnancy	1.00	1.35 (1.10-1.65)	1.00	0.84 (0.64-1.12)	1.51 (1.09-2.09)	1.00 (0.69-1.45)
Antibiotic use in pregnancy	1.00	1.33 (1.08-1.63)	1.00	0.84 (0.64-1.12)	1.49 (1.08-2.06)	0.98 (0.68-1.42)

ADHD: attention deficit hyperactivity disorder, SES: Socioeconomic status, IMD: Index of Multiple Deprivation, BMI: body mass index.

* n = 7,165

† n = 4,961

sTable 5. Odds ratio (95% confidence interval) for attention deficit hyperactivity disorder according to asthma status or asthma endotypes, all at 7 years, cumulatively adjusted for shared risk factors.

	**Asthma***		**Asthma endotypes†**			
	**No**	**Yes**	**None**	**Atopy without asthma**	**Nonatopic asthma**	**Atopic asthma**
**ADHD** Cases/non-cases	633/5562	129/841	359/3204	63/666	48/284	34/303
Crude	1.00	1.35 (1.10-1.65)	1.00	0.84 (0.64-1.12)	1.51 (1.09-2.09)	1.00 (0.69-1.45)
**Adjusted for**						
Sex	1.00	1.28 (1.04-1.57)	1.00	0.78 (0.59-1.04)	1.46 (1.05-2.02)	0.91 (0.63-1.33)
+ All 4 SES factors	1.00	1.21 (0.98-1.49)	1.00	0.81 (0.61-1.08)	1.32 (0.94-1.85)	0.92 (0.63-1.34)
+ Anxiety score	1.00	1.17 (0.95-1.44)	1.00	0.81 (0.61-1.08)	1.26 (0.90-1.77)	0.90 (0.61-1.31)
+ Depression score	1.00	1.17 (0.95-1.45)	1.00	0.82 (0.62-1.09)	1.28 (0.91-1.80)	0.90 (0.61-1.31)
+ Gestational age	1.00	1.17 (0.95-1.44)	1.00	0.83 (0.62-1.10)	1.26 (0.90-1.77)	0.90 (0.62-1.32)
+ Smoking in pregnancy	1.00	1.17 (0.95-1.44)	1.00	0.84 (0.63-1.12)	1.26 (0.90-1.77)	0.90 (0.62-1.32)
+ Paracetamol	1.00	1.16 (0.94-1.43)	1.00	0.84 (0.63-1.12)	1.25 (0.89-1.76)	0.90 (0.62-1.32)
+ Maternal age	1.00	1.16 (0.94-1.43)	1.00	0.84 (0.63-1.12)	1.25 (0.89-1.76)	0.90 (0.61-1.31)
+ Birth weight	1.00	1.15 (0.93-1.42)	1.00	0.84 (0.63-1.12)	1.23 (0.88-1.73)	0.90 (0.61-1.31)
+ Preeclampsia	1.00	1.15 (0.93-1.42)	1.00	0.84 (0.63-1.12)	1.23 (0.88-1.73)	0.90 (0.61-1.31)
+ Free sugar & total energy intake	1.00	1.15 (0.93-1.42)	1.00	0.83 (0.62-1.11)	1.25 (0.89-1.75)	0.89 (0.61-1.30)
+ BMI before pregnancy	1.00	1.15 (0.93-1.42)	1.00	0.83 (0.62-1.11)	1.24 (0.88-1.75)	0.89 (0.61-1.30)
+ Antibiotic use in pregnancy	1.00	1.14 (0.92-1.41)	1.00	0.83 (0.62-1.11)	1.24 (0.88-1.74)	0.87 (0.59-1.28)

ADHD: attention deficit hyperactivity disorder, SES: Socioeconomic status, IMD: Index of Multiple Deprivation, BMI: body mass index.
* n = 7,165
† n = 4,961

sTable 6. Odds ratio (95% confidence interval) for attention deficit hyperactivity disorder at 9 years according to asthma status or asthma endotypes at 7 years, cumulatively adjusted for shared risk factors.

	**Asthma***		**Asthma endotypes†**			
	**No**	**Yes**	**None**	**Atopy without asthma**	**Nonatopic asthma**	**Atopic asthma**
**ADHD** Cases/non-cases	418/5369	91/825	239/3187	40/660	28/285	27/304
Crude	1.00	1.42 (1.12-1.80)	1.00	0.81 (0.57-1.14)	1.31 (0.87-1.97)	1.18 (0.78-1.79)
**Adjusted for**						
Sex	1.00	1.32 (1.04-1.68)	1.00	0.75 (0.53-1.06)	1.24 (0.82-1.87)	1.09 (0.72-1.65)
+ All 4 SES factors	1.00	1.26 (0.99-1.61)	1.00	0.79 (0.56-1.12)	1.13 (0.74-1.73)	1.10 (0.72-1.68)
+ Anxiety score	1.00	1.20 (0.94-1.54)	1.00	0.79 (0.55-1.12)	1.06 (0.69-1.62)	1.08 (0.71-1.65)
+ Depression score	1.00	1.21 (0.95-1.55)	1.00	0.79 (0.56-1.13)	1.06 (0.69-1.63)	1.08 (0.71-1.65)
+ Gestational age	1.00	1.21 (0.94-1.54)	1.00	0.80 (0.56-1.14)	1.05 (0.68-1.61)	1.08 (0.71-1.65)
+ Smoking in pregnancy	1.00	1.21 (0.95-1.55)	1.00	0.81 (0.57-1.16)	1.05 (0.69-1.61)	1.08 (0.71-1.65)
+ Paracetamol	1.00	1.20 (0.94-1.53)	1.00	0.82 (0.58-1.17)	1.04 (0.68-1.59)	1.07 (0.70-1.64)
+ Maternal age	1.00	1.20 (0.94-1.53)	1.00	0.82 (0.58-1.17)	1.04 (0.68-1.59)	1.07 (0.70-1.64)
+ Birth weight	1.00	1.19 (0.93-1.52)	1.00	0.82 (0.57-1.16)	1.03 (0.67-1.58)	1.07 (0.70-1.63)
+ Preeclampsia	1.00	1.19 (0.93-1.52)	1.00	0.81 (0.57-1.16)	1.04 (0.67-1.59)	1.07 (0.70-1.64)
+ Free sugar & total energy intake	1.00	1.18 (0.92-1.51)	1.00	0.80 (0.56-1.15)	1.04 (0.68-1.61)	1.04 (0.68-1.60)
+ BMI before pregnancy	1.00	1.18 (0.92-1.51)	1.00	0.81 (0.57-1.15)	1.03 (0.67-1.59)	1.04 (0.68-1.60)
+ Antibiotic use in pregnancy	1.00	1.18 (0.92-1.51)	1.00	0.81 (0.57-1.15)	1.04 (0.67-1.60)	1.05 (0.68-1.61)

ADHD: attention deficit hyperactivity disorder, SES: Socioeconomic status, IMD: Index of Multiple Deprivation, BMI: body mass index.
* n = 6,703
† n = 4,770

sTable 7. The association of polygenic risk scores for asthma with asthma at 7 years and indicators of goodness of fit.

	**OR (95% CI) per Z-score**	**AIC**	**BIC**	**Nagelkerke pseudo R^2**	**AUC**
**Asthma PRS (*P*-value thresholds)**					
S1: 5×10^{-1}	2.14 (1.97-2.32)	3881	3961	0.0649	0.492
S2: 1×10^{-1}	2.07 (1.90-2.25)	3913	3993	0.0594	0.497
S3: 5×10^{-2}	1.97 (1.81-2.14)	3955	4034	0.0521	0.495
S4: 1×10^{-2}	1.73 (1.59-1.87)	4057	4136	0.0342	0.499
S5: 5×10^{-3}	1.66 (1.53-1.80)	4081	4160	0.0299	0.510
S6: 1×10^{-3}	1.47 (1.36-1.59)	4145	4225	0.0183	0.505
S7: 5×10^{-8}	1.26 (1.16-1.36)	4204	4283	0.0077	0.484

N = 5,425

OR: Odds Ratio, CI: confidence interval, AIC: Akaike information criterion, BIC: Bayesian information criterion, AUC: Area under the Curve.

sTable 8. The association of polygenic risk scores for asthma with asthma endotypes at 7 years and indicators of goodness of fit.

Asthma PRS (*P* thresholds)	None	Atopy without asthma	Nonatopic asthma	Atopic asthma	AIC	BIC	Nagelkerke pseudo R^2	AUC
		RRR (95% CI)	RRR (95% CI)	RRR (95% CI)				
S1: 5×10^{-1}	1.00	1.07 (0.99-1.17)	2.43 (2.14-2.77)	2.55 (2.24-2.90)	6759	6986	0.0971	0.505
S2: 1×10^{-1}	1.00	1.07 (0.99-1.17)	2.29 (2.01-2.60)	2.44 (2.15-2.78)	6805	7033	0.0868	0.501
S3: 5×10^{-2}	1.00	1.07 (0.98-1.17)	2.17 (1.91-2.47)	2.23 (1.96-2.53)	6861	7089	0.0742	0.505
S4: 1×10^{-2}	1.00	1.07 (0.97-1.16)	1.79 (1.57-2.04)	2.05 (1.80-2.33)	6957	7184	0.0523	0.501
S5: 5×10^{-3}	1.00	1.06 (0.97-1.15)	1.73 (1.52-1.97)	1.93 (1.70-2.20)	6982	7210	0.0464	0.490
S6: 1×10^{-3}	1.00	1.06 (0.97-1.16)	1.34 (1.18-1.52)	1.86 (1.63-2.11)	7037	7265	0.0335	0.495
S7: 5×10^{-8}	1.00	1.03 (0.94-1.12)	1.22 (1.08-1.39)	1.45 (1.28-1.65)	7103	7330	0.0178	0.514

N = 4,089

Multinomial logistic regression models were used.

RRR: Relative Risk Ratio, CI: confidence interval, AIC: Akaike information criterion, BIC: Bayesian information criterion, AUC: Area under the Curve.

sTable 9. Association of polygenic risk scores for attention deficit hyperactivity disorder (ADHD) with ADHD at 7 years and indicators of goodness of fit.

	**OR (95% CI) per Z-score**	**AIC**	**BIC**	**Nagelkerke pseudo R^2**	**AUC**
**ADHD PRS (*P*-value thresholds)**					
S1: 5×10^{-1}	1.14 (1.04-1.24)	3653	3732	0.0032	0.551
S2: 1×10^{-1}	1.15 (1.05-1.25)	3651	3731	0.0034	0.555
S3: 5×10^{-2}	1.16 (1.06-1.26)	3651	3730	0.0035	0.556
S4: 1×10^{-2}	1.18 (1.08-1.28)	3648	3727	0.0041	0.560
S5: 5×10^{-3}	1.18 (1.08-1.29)	3648	3727	0.0041	0.560
S6: 1×10^{-3}	1.19 (1.09-1.30)	3646	3726	0.0044	0.561
S7: 5×10^{-8}	1.02 (0.94-1.12)	3661	3740	0.0017	0.538

N = 5,503

OR: Odds Ratio, CI: confidence interval, AIC: Akaike information criterion, BIC: Bayesian information criterion, AUC: Area under the Curve.

sTable 10. Associations of polygenic risk scores for asthma with attention deficit hyperactivity disorder at 7 years and polygenic risk scores for attention deficit hyperactivity disorder with asthma at 7 years.

	**Outcome**			
***P*-value thresholds**	**No**	**Yes***	***P*-value**	**Variance Explained (%)†**
**ADHD**, n (%)	4,937 (89.7)	566 (10.3)		
**Asthma PRS**				
5×10^{-1}	1.00	0.97 (0.89-1.05)	0.45	1.38
1×10^{-1}	1.00	0.98 (0.90-1.06)	0.59	1.38
5×10^{-2}	1.00	0.98 (0.90-1.07)	0.61	1.38
1×10^{-2}	1.00	1.00 (0.91-1.09)	0.92	1.37
5×10^{-3}	1.00	1.04 (0.95-1.13)	0.42	1.39
1×10^{-3}	1.00	1.02 (0.94-1.11)	0.63	1.38
5×10^{-8}	1.00	0.97 (0.89-1.06)	0.45	1.38
**Asthma**, n (%)	4,712 (86.9)	713 (13.1)		
**ADHD PRS**				
5×10^{-1}	1.00	1.08 (1.00-1.17)	0.05	0.62
1×10^{-1}	1.00	1.09 (1.01-1.18)	0.03	0.63
5×10^{-2}	1.00	1.10 (1.02-1.19)	0.02	0.65
1×10^{-2}	1.00	1.10 (1.02-1.19)	0.02	0.65
5×10^{-3}	1.00	1.12 (1.04-1.22)	0.004	0.70
1×10^{-3}	1.00	1.10 (1.02-1.19)	0.02	0.65
5×10^{-8}	1.00	1.02 (0.94-1.11)	0.59	0.56

N = 5,503 (asthma PRSs-ADHD) and 5,425 (ADHD PRSs-asthma).

PRS: polygenic risk score; ADHD: attention deficit hyperactivity disorder

* Associations are presented as odds ratio per Z score (95% confidence interval), adjusted for sex and the first 10 genetic principal components.

† Variance explained on liability scale (%) based on Nagelkerke pseudo R^2 in adjusted models.

sTable 11. Association of polygenic risk scores for attention deficit hyperactivity disorder (ADHD) with ADHD at 9 years and indicators of goodness of fit.

	**OR (95% CI) per Z-score**	**AIC**	**BIC**	**Nagelkerke pseudo R^2**	**AUC**
**ADHD PRS (*P*-value thresholds)**					
S1: $5×10^{-1}$	1.25 (1.13-1.39)	2856	2935	0.0123	0.543
S2: $1×10^{-1}$	1.26 (1.14-1.40)	2855	2934	0.0128	0.545
S3: $5×10^{-2}$	1.27 (1.15-1.41)	2853	2932	0.0135	0.546
S4: $1×10^{-2}$	1.25 (1.13-1.39)	2856	2935	0.0124	0.551
S5: $5×10^{-3}$	1.23 (1.11-1.37)	2858	2937	0.0113	0.550
S6: $1×10^{-3}$	1.23 (1.11-1.37)	2858	2937	0.0115	0.551
S7: $5×10^{-8}$	1.07 (0.97-1.18)	2873	2951	0.0049	0.528

N = 5,288

OR: Odds Ratio, CI: confidence interval, AIC: Akaike information criterion, BIC: Bayesian information criterion, AUC: Area under the Curve.

sTable 12. Association of polygenic risk score for asthma with attention deficit hyperactivity disorder at 9 years.

	ADHD at 9 years		*P*-value	Variance Explained (%)[†]
	**No**	**Yes**[*]		
**ADHD**, n (%)	4,883 (92.3)	405 (7.7)		
**Asthma PRS (*P*-value thresholds)**				
S1: 5×10^{-1}	1.00	1.00 (0.91-1.11)	0.96	1.33
S2: 1×10^{-1}	1.00	1.01 (0.91-1.11)	0.86	1.33
S3: 5×10^{-2}	1.00	1.00 (0.90-1.10)	0.98	1.33
S4: 1×10^{-2}	1.00	1.04 (0.94-1.15)	0.42	1.34
S5: 5×10^{-3}	1.00	1.06 (0.96-1.17)	0.26	1.35
S6: 1×10^{-3}	1.00	0.97 (0.87-1.07)	0.52	1.33
S7: 5×10^{-8}	1.00	0.96 (0.87-1.06)	0.45	1.34

N = 5,288

PRS: Polygenic Risk Score; ADHD: attention deficit hyperactivity disorder

[*] Associations are presented as odds ratio per Z score (95% confidence interval), adjusted for sex and the first 10 genetic principal components

[†] Variance Explained on Liability Scale (%) based on Nagelkerke pseudo R^2 in adjusted models.

sTable 13. Association of polygenic risk score for eczema and hay fever with attention deficit hyperactivity disorder at 7 years of age.

	ADHD			
	**No**	**Yes***	***P*-value**	**Variance Explained (%)†**
n (%)	4,937 (89.7)	566 (10.3)		
***P*-value thresholds for eczema PRS**				
$5×10^{-1}$	1.00	1.11 (1.00-1.23)	0.04	1.45
$1×10^{-1}$	1.00	1.08 (0.98-1.18)	0.12	1.42
$5×10^{-2}$	1.00	1.08 (0.99-1.18)	0.10	1.42
$1×10^{-2}$	1.00	1.06 (0.98-1.16)	0.16	1.41
$5×10^{-3}$	1.00	1.03 (0.95-1.13)	0.46	1.38
$1×10^{-3}$	1.00	0.99 (0.90-1.07)	0.75	1.38
$5×10^{-8}$	1.00	0.90 (0.82-0.98)	0.02	1.47
***P*-value thresholds for hay fever PRS**				
$5×10^{-1}$	1.00	0.97 (0.89-1.06)	0.53	1.38
$1×10^{-1}$	1.00	0.96 (0.89-1.05)	0.40	1.39
$5×10^{-2}$	1.00	0.96 (0.89-1.05)	0.38	1.39
$1×10^{-2}$	1.00	0.95 (0.87-1.03)	0.20	1.40
$5×10^{-3}$	1.00	0.96 (0.88-1.05)	0.37	1.39
$1×10^{-3}$	1.00	0.99 (0.91-1.08)	0.86	1.37
$5×10^{-8}$	1.00	0.99 (0.91-1.08)	0.90	1.37
***P*-value thresholds for allergic sensitisation PRS**				
$5×10^{-1}$	1.00	0.94 (0.86-1.03)	0.18	1.41
$1×10^{-1}$	1.00	0.95 (0.88-1.04)	0.27	1.40
$5×10^{-2}$	1.00	0.95 (0.87-1.04)	0.25	1.40
$1×10^{-2}$	1.00	0.95 (0.87-1.03)	0.23	1.40
$5×10^{-3}$	1.00	0.95 (0.87-1.03)	0.20	1.40
$1×10^{-3}$	1.00	0.97 (0.89-1.06)	0.56	1.38
$5×10^{-8}$	1.00	1.01 (0.93-1.11)	0.75	1.38

N = 5,503

PRS: Polygenic Risk Score; ADHD: attention deficit hyperactivity disorder

* Associations are presented as odds ratio per Z score (95% confidence interval), adjusted for sex and the first 10 genetic principal components.

† Variance Explained on Liability Scale (%) based on Nagelkerke pseudo R^2 in adjusted models.

sTable 14. Association of polygenic risk score for attention deficit hyperactivity disorder with eczema and hay fever at 7 years of age.

	No	Yes*	*P*-value	Variance Explained (%)†
**Eczema,** n (%)	4,566 (86.9)	901 (16.5)		
***P*-value thresholds for ADHD PRS**				
$5{\times}10^{-1}$	1.00	1.01 (0.94-1.09)	0.78	0.41
$1{\times}10^{-1}$	1.00	1.03 (0.96-1.11)	0.40	0.42
$5{\times}10^{-2}$	1.00	1.02 (0.95-1.09)	0.61	0.41
$1{\times}10^{-2}$	1.00	1.01 (0.94-1.08)	0.82	0.41
$5{\times}10^{-3}$	1.00	1.01 (0.94-1.08)	0.86	0.41
$1{\times}10^{-3}$	1.00	1.02 (0.95-1.09)	0.65	0.41
$5{\times}10^{-8}$	1.00	1.04 (0.97-1.12)	0.29	0.43
**Hay fever,** n (%)	4,982 (91.4)	469 (8.6)		
***P*-value thresholds for ADHD PRS**				
$5{\times}10^{-1}$	1.00	0.91 (0.83-1.00)	0.06	0.77
$1{\times}10^{-1}$	1.00	0.92 (0.84-1.01)	0.09	0.75
$5{\times}10^{-2}$	1.00	0.92 (0.83-1.01)	0.08	0.76
$1{\times}10^{-2}$	1.00	0.94 (0.86-1.04)	0.23	0.73
$5{\times}10^{-3}$	1.00	0.97 (0.88-1.07)	0.52	0.71
$1{\times}10^{-3}$	1.00	0.97 (0.88-1.06)	0.49	0.71
$5{\times}10^{-8}$	1.00	1.00 (0.91-1.10)	0.93	0.70
**Atopy,** n (%)	3725 (78.9)	996 (21.1)		
***P*-value thresholds for ADHD PRS**				
$5{\times}10^{-1}$	1.00	0.98 (0.92-1.06)	0.64	1.02
$1{\times}10^{-1}$	1.00	0.97 (0.91-1.05)	0.47	1.02
$5{\times}10^{-2}$	1.00	0.99 (0.92-1.06)	0.70	1.02
$1{\times}10^{-2}$	1.00	0.96 (0.89-1.03)	0.21	1.05
$5{\times}10^{-3}$	1.00	0.98 (0.91-1.05)	0.56	1.02
$1{\times}10^{-3}$	1.00	0.97 (0.91-1.04)	0.42	1.03
$5{\times}10^{-8}$	1.00	1.04 (0.97-1.11)	0.33	1.03

N = 5,467 (eczema); 5,451 (hay fever); and 4,721 (atopy)

PRS: Polygenic Risk Score; ADHD: attention deficit hyperactivity disorder

* Associations are presented as odds ratio per Z score (95% confidence interval), adjusted for sex and the first 10 genetic principal components.

† Variance Explained on Liability Scale (%) based on Nagelkerke pseudo R^2 in adjusted models.

sTable 15. Characteristics of 19 genetic variants associated with genetic liability to asthma.

RS ID	Chr: Position	Effect allele / other allele	Effect size (SE)	*P*-value
rs3771180	2:102953617	G/T	0.1742 (0.0187)	1.47E-20
rs6893213	5:110198114	T/C	0.1452 (0.0223)	7.99E-11
rs10455025	5:110404999	C/A	0.1402 (0.0134)	2.03E-25
rs6894249	5:131797547	G/A	0.0873 (0.0130)	2.15E-11
rs20541	5:131995964	A/G	0.1196 (0.0155)	1.36E-14
rs7705042	5:141492419	A/C	0.0795 (0.0129)	8.53E-10
rs2523454	6:31367865	A/G	0.0890 (0.0136)	6.05E-11
rs9272346	6:32604372	A/G	0.1470 (0.0133)	2.35E-28
rs2325291	6:90986686	G/A	0.0955 (0.0133)	8.58E-13
rs10957979	8:81289787	A/G	0.0739 (0.0132)	2.33E-08
rs992969	9:6209697	A/G	0.1581 (0.0141)	4.27E-29
rs1663687	10:9054787	G/A	0.0843 (0.0131)	1.52E-10
rs2155219	11:76299194	T/G	0.1050 (0.0132)	2.90E-15
rs167769	12:57503775	T/C	0.0759 (0.0130)	5.50E-09
rs10519067	15:61068347	G/A	0.1208 (0.0188)	1.49E-10
rs17293632	15:67442596	T/C	0.1179 (0.0146)	8.81E-16
rs12935657	16:11219041	G/A	0.1037 (0.0147)	2.06E-12
rs2305479	17:38062217	C/T	0.1782 (0.0130)	1.00E-42
rs17637472	17:47461433	A/G	0.0776 (0.0131)	3.28E-09

sTable 16. Characteristics of 13 genetic variants associated with genetic liability to atopic dermatitis (eczema).

RS ID	Chr: Position	Effect allele / other allele	Effect size (SE)	*P*-value
rs2477121	1:150290762	T/A	0.0996 (0.0175)	1.45E-08
rs12144049	1:152440910	C/T	0.2018 (0.0186)	2.80E-27
rs61815704	1:152893891	G/C	0.5425 (0.0589)	3.91E-20
rs6419573	2:103027103	T/C	0.1239 (0.0196)	2.92E-10
rs12188917	5:131991085	C/T	0.1700 (0.0215)	2.89E-15
rs4151657	6:31917540	C/T	0.1019 (0.0176)	7.86E-09
rs12334935	8:126617990	A/G	0.0926 (0.0168)	4.18E-08
rs10790275	11:118745884	C/G	0.1224 (0.0218)	2.16E-08
rs479844	11:65551957	G/A	0.1437 (0.0170)	3.45E-17
rs2212434	11:76281593	T/C	0.1291 (0.0168)	2.09E-14
rs8066625	17:40390629	A/G	0.1755 (0.0319)	3.84E-08
rs2918299	19:8787273	T/C	0.1425 (0.0229)	5.45E-10
rs6062486	20:62302539	A/G	0.1045 (0.0187)	2.40E-08

sTable 17. Characteristics of 3 genetic variants associated with genetic liability to Atopic rhinitis / Hay fever.

RS ID	Chr: Position	Effect allele / other allele	Effect size (SE)	*P*-value
rs67719080	4:38787252	A/G	0.1219 (0.022)	3.73E-08
rs114176380	6:33437236	A/G	0.2539 (0.045)	2.11E-08
rs7936070	11:76293527	T/G	0.1448 (0.0184)	4.86E-15

sTable 18. Characteristics of 11 genetic variants associated with genetic liability to allergic sensitisation (atopy).

RS ID	Chr: Position	Effect allele / other allele	Effect size (SE)	*P*-value
rs78037977	1:172715702	A/G	0.2108 (0.0344)	9.19E-10
rs2160203	2:102960824	A/G	0.1445 (0.0244)	3.37E-09
rs891058	2:8442547	G/A	0.1313 (0.0231)	1.27E-08
rs9865818	3:188072513	G/A	0.1275 (0.0216)	3.84E-09
rs17616434	4:38812876	T/C	0.1930 (0.0263)	2.23E-13
rs2069772	4:123373133	C/T	0.1304 (0.0229)	1.36E-08
rs4648050	4:103514741	T/C	0.1262 (0.0225)	2.03E-08
rs12657787	5:110187054	A/G	0.1910 (0.0293)	6.90E-11
rs1379298	5:110435726	T/C	0.1183 (0.0213)	2.95E-08
rs7936070	11:76293527	T/G	0.1766 (0.0231)	2.11E-14
rs1059513	12:57489709	T/C	0.2333 (0.0383)	1.18E-09

sTable 19. Characteristics of 26 genetic variants associated with genetic liability to attention deficit hyperactivity disorder (ADHD).

RS ID	Chr: Position	Effect allele / other allele	Effect size (SE)	*P*-value
rs549845	1:44076469	G/A	0.0788 (0.0102)	9.03E-15
rs1438898	2:145714354	A/C	0.0629 (0.0108)	4.88E-09
rs2886697	3:20724204	G/A	0.0588 (0.0096)	7.90E-10
rs114142727	3:87015142	C/G	0.2506 (0.0403)	5.13E-10
rs115111850	3:43651029	G/A	0.1149 (0.0204)	1.71E-08
rs17718444	3:71499401	C/T	0.0612 (0.0103)	2.87E-09
rs2311059	3:51884072	A/G	0.0581 (0.0105)	3.16E-08
rs6537401	4:147099654	A/G	0.0568 (0.0100)	1.40E-08
rs17576773	4:112217523	C/T	0.0963 (0.0151)	1.63E-10
rs77960	5:103964585	A/G	0.0732 (0.0100)	2.46E-13
rs10875612	5:144474779	T/C	0.0543 (0.0093)	5.62E-09
rs4916723	5:87854395	C/A	0.0853 (0.0110)	9.48E-15
rs2025286	6:70858701	C/A	0.0549 (0.0093)	4.00E-09
rs9969232	7:114158954	A/G	0.0683 (0.0100)	9.98E-12
rs73145587	7:67685754	A/T	0.1013 (0.0184)	3.67E-08
rs4925811	8:145802447	G/T	0.0580 (0.0101)	8.30E-09
rs7844069	8:93277087	T/G	0.0552 (0.0095)	6.74E-09
rs11255890	10:8784773	C/A	0.0530 (0.0097)	4.14E-08
rs11596214	10:106453832	G/A	0.0528 (0.0095)	3.17E-08
rs2582895	11:28602173	C/A	0.0725 (0.0096)	4.09E-14
rs704061	12:89771903	C/T	0.0559 (0.0094)	2.30E-09
rs76284431	14:98690923	A/T	0.0816 (0.0134)	1.19E-09
rs1162202	16:61966703	C/T	0.0614 (0.0102)	1.92E-09
rs76857496	18:5871800	C/A	0.0800 (0.0140)	1.24E-08
rs7506904	18:50625779	A/G	0.0559 (0.0098)	1.24E-08
rs6082363	20:21250843	T/C	0.0703 (0.0101)	4.38E-12

sTable 20. Bidirectional causal effect estimates of genetic liability to asthma and ADHD as estimated by IVW, MR-Egger and weighted median estimator.

**Method**	**No. SNPs**	**OR**	**95% CI**	***P*-value**	**Q *P*-value**
**Causal effect of genetic liability to asthma on ADHD**					
IVW	19	1.00	(0.94-1.06)	0.89	0.35
MR – Egger					
Intercept	19	1.02	(1.00-1.04)	0.09	
Slope	19	0.84	(0.69-1.03)	0.10	0.46
Weighted Median	19	0.97	(0.89-1.05)	0.45	
**Causal effect of genetic liability to ADHD on asthma**					
IVW	20*	1.06	(0.92-1.23)	0.41	4.45×10^{-04}
MR – Egger					
Intercept	20*	1.04	(0.99-1.09)	0.08	
Slope	20*	0.56	(0.27-1.17)	0.12	29×10^{-03}
Weighted Median	20*	1.05	(0.90-1.21)	0.54	

* Genetic variant (*rs73145587*) was excluded due to being palindromic.
ADHD: attention deficit hyperactivity disorder, OR: Odds ratio, CI: confidence interval, IVW: inverse variance weighted, MR-Egger: Mendelian randomisation-egger.

sTable 21. Bidirectional causal effect estimates of genetic liability to atopic dermatitis (eczema) and as estimated by IVW, MR-Egger and weighted median estimator.

Method	No. SNPs	OR	95% CI	*P*-value	Q *P*-value
**Causal effect of genetic liability to eczema on ADHD**					
IVW	10	1.07	(1.00-1.15)	0.05	0.72
MR – Egger					
Intercept	10	0.99	(0.95-1.03)	0.48	
Slope	10	1.18	(0.89-1.57)	0.25	0.68
Weighted Median	10	1.12	(1.02-1.22)	0.02	
**Causal effect of genetic liability to ADHD on eczema**					
IVW	8	0.88	(0.76-1.03)	0.11	0.48
MR – Egger					
Intercept	8	1.03	(0.96-1.09)	0.41	
Slope	8	0.66	(0.32-1.35)	0.26	0.44
Weighted Median	8	0.93	(0.77-1.15)	0.55	
Weighted Mode	8	0.99	(0.75-1.32)	0.97	

ADHD: attention deficit hyperactivity disorder, OR: Odds ratio, CI: confidence interval, IVW: inverse variance weighted, MR-Egger: Mendelian randomisation-egger.

sTable 22. Bidirectional causal effect estimates of genetic liability to atopic rhinitis (hay fever) and ADHD as estimated by IVW, MR-Egger and weighted median estimator.

**Method**	**No. SNPs**	**OR**	**95% CI**	***P*-value**	**Q *P*-value**
**Causal effect of genetic liability to hay fever on ADHD**					
IVW	3	1.12	(0.96-1.32)	0.14	0.23
MR – Egger					
Intercept	3	0.95	(0.86-1.04)	0.28	
Slope	3	1.58	(0.84-2.96)	0.16	0.24
Weighted Median	3	1.18	(1.01-1.38)	0.04	
**Causal effect of genetic liability to ADHD on hay fever**					
IVW	8	0.91	(0.78-1.06)	0.23	0.82
MR – Egger					
Intercept	8	1.00	(0.94-1.06)	<0.99	
Slope	8	0.91	(0.46-1.80)	0.79	0.73
Weighted Median	8	0.92	(0.76-1.12)	0.42	

ADHD: attention deficit hyperactivity disorder, OR: Odds ratio, CI: confidence interval, IVW: inverse variance weighted, MR-Egger: Mendelian randomisation-egger.

sTable 23. Bidirectional causal effect estimates of genetic liability to allergic sensitisation and ADHD as estimated by IVW, MR-Egger and weighted median estimator.

Method	No. SNPs	OR	95% CI	*P*-value	Q *P*-value
**Causal effect of genetic liability to allergic sensitisation on ADHD**					
IVW	11	1.00	(0.91-1.09)	0.94	0.01
MR – Egger					
Intercept	11	1.01	(0.94-1.08)	0.82	
Slope	11	0.95	(0.61-1.47)	0.81	0.01
Weighted Median	11	0.97	(0.89-1.06)	0.55	
**Causal effect of genetic liability to ADHD on allergic sensitisation**					
IVW	8	0.91	(0.76-1.10)	0.31	0.88
MR – Egger					
Intercept	8	0.96	(0.89-1.04)	0.30	
Slope	8	1.39	(0.60-3.18)	0.44	0.91
Weighted Median	8	0.90	(0.71-1.14)	0.39	
Weighted Mode	8	0.92	(0.65-1.29)	0.62	

ADHD: attention deficit hyperactivity disorder, OR: Odds ratio, CI: confidence interval, IVW: inverse variance weighted, MR-Egger: Mendelian randomisation-egger.

sTable 24. Factors that the association between asthma and ADHD were controlled for in previous studies.*

	Yang, 2018	Wang, 2018	Chen, 2017	Jameson, 2016	Kline-Simon, 2016	van der Schans, 2016	Lin, 2016	Strom, 2016	Turan Akyol, 2015	Silva, 2014	James, 2013	Hak, 2013	Goodwin, 2013	Suwan, 2011	Romanos, 2010	Van Den Heuvel, 2007	Alabaf, 2019	Kwon, 2014	Karlstad, 2012	Saricoban, 2011	Meyers, 2010	Leibson, 2001	Biederman, 1995	Flannery, 1995
Age	✓	✓	✓	✓	✓	✓	✓	✓	✓	✓	✓	✓		✓		✓								
sex	✓	✓	✓	✓	✓	✓	✓	✓		✓	✓			✓		✓								
Race/ethnicity				✓				✓																
Region						✓	✓	✓				✓			✓									
Family characteristics							✓			✓			✓		✓									
Family income							✓	✓							✓									
Insurance coverage								✓																
Maternal education	✓			✓				✓					✓											
Socioeconomic status index (area)										✓					✓									
Maternal history of atopy	✓														✓									
Eczema	✓					✓		✓				✓												
Allergic rhinitis						✓						✓												
Height		✓																						
Weight		✓									✓													
Psychiatric comorbidities			✓																					
Maternal history of mental health problems													✓											
Other disorders				✓		✓									✓									
Medical Facility					✓						✓													
Exposure to tobacco at home							✓		✓				✓		✓									
Birth weight	✓									✓		✓												
Breastfeeding	✓														✓									
Maternal smoking during pregnancy							✓						✓		✓									
Preterm status							✓			✓		✓												
Maternal age										✓			✓											

* Included in meta-analysis by Kaas TH, et al. Clin Exp Allergy. 2021;51(2):228-52.

Socioeconomic status factors and early life risk factors were highlighted in amber and orange, respectively.
